# Unveiling the Accelerated Water Electrolysis Kinetics of Heterostructural Iron‐Cobalt‐Nickel Sulfides by Probing into Crystalline/Amorphous Interfaces in Stepwise Catalytic Reactions

**DOI:** 10.1002/advs.202201903

**Published:** 2022-09-04

**Authors:** Zhengxiang Gu, Yechuan Zhang, Xuelian Wei, Zhenyu Duan, Long Ren, Jiecheng Ji, Xiaoqin Zhang, Yuxin Zhang, Qiyong Gong, Hao Wu, Kui Luo

**Affiliations:** ^1^ Huaxi MR Research Center (HMRRC) Animal Experimental Center Department of Radiology National Clinical Research Center for Geriatrics Frontiers Science Center for Disease‐Related Molecular Network State Key Laboratory of Biotherapy West China Hospital Sichuan University Chengdu 610041 P. R. China; ^2^ School of Chemical Engineering and Advanced Materials University of Adelaide Adelaide SA 5005 Australia; ^3^ National Engineering Research Center for Biomaterials Sichuan University 29 Wangjiang Road Chengdu 610064 P. R. China; ^4^ Functional and Molecular Imaging Key Laboratory of Sichuan Province and Research Unit of Psychoradiology Chinese Academy of Medical Sciences Chengdu 610041 P. R. China; ^5^ Institute of Molecular Sciences and Engineering Institute of Frontier and Interdisciplinary Science Shandong University Qingdao Shandong 266237 P. R. China

**Keywords:** crystalline/amorphous interfaces, electrocatalysis, heterostructures, water splitting

## Abstract

Amorphization and crystalline grain boundary engineering are adopted separately in improving the catalytic kinetics for water electrolysis. Yet, the synergistic effect and advance in the cooperated form of crystalline/amorphous interfaces (CAI) have rarely been elucidated insightfully. Herein, a trimetallic FeCo(NiS_2_)_4_ catalyst with numerous CAI (FeCo(NiS_2_)_4_‐C/A) is presented, which shows highly efficient catalytic activity toward both hydrogen and oxygen evolution reactions (HER and OER). Density functional theory (DFT) studies reveal that CAI plays a significant role in accelerating water electrolysis kinetics, in which Co atoms on the CAI of FeCo(NiS_2_)_4_‐C/A catalyst exhibit the optimal binding energy of 0.002 eV for H atoms in HER while it also has the lowest reaction barrier of 1.40 eV for the key step of OER. H_2_O molecules are inclined to be absorbed on the interfacial Ni atoms based on DFT calculations. As a result, the heterostructural CAI‐containing catalyst shows a low overpotential of 82 and 230 mV for HER and OER, respectively. As a bifunctional catalyst, it delivers a current density of 10 mA cm^−2^ at a low cell voltage of 1.51 V, which enables it a noble candidate as metal‐based catalysts for water splitting. This work explores the role of CAI in accelerating the HER and OER kinetics for water electrolysis, which sheds light on the development of efficient, stable, and economical water electrolysis systems by facile interface‐engineering implantations.

## Graphical abstract

The FeCo(NiS_2_)_4_‐C/A electrocatalyst with sufficient crystalline/amorphous interfaces is designed and prepared as a bifunctional electrode, showing a low overpotential of 82 and 230 mV for hydrogen and oxygen evolution reactions, respectively.

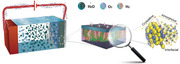

## 1 Introduction

The exploration of highly active catalysts for water electrolysis (hydrogen and oxygen evolution reactions, HER and OER) is vital to the sustainable production of high‐purity hydrogen and oxygen. In pursuit of kinetics‐favorable catalysts beyond scarce noble metals and compounds, the activation of electrochemically passive catalysts provides a promising solution. Amorphization,^[^ ^1^, ^2^ ^]^ defect engineering,^[^ ^3^, ^4^ ^]^ phase engineering,^[^ ^5^, ^6^ ^]^ downsizing,^[^ ^7^, ^8^ ^]^ heterostructure construction,^[^ ^9^, ^10^ ^]^ and grain boundary engineering^[^ ^11^ ^]^ have been involved in the design strategies to achieve this goal. Amorphization, among these methods, is capable of improving the catalytic activity due to the increased active sites, rich surface defects, and available dangling bonds in the amorphous phase. Intrinsically, because the lattice distortion and surface dangling bonds in the amorphous phase could favor the generation of active *OOH intermediates of OER, the amorphous counterparts could exhibit superior activity. For instance, Indra et al.^[^ ^12^ ^]^ reported that amorphous cobalt iron oxide exhibits better OER performance than their crystalline counterparts under the same electrochemical environment. Liu et al.^[^ ^13^ ^]^ illustrated that amorphous cobalt could contribute to the transformation of its ultrathin, amorphous and alloyed structure into active phases, leading to optimized electrocatalytic performance for OER. Yet, the poor crystallinity and defective structure of amorphous material would induce high solubility and poor stability in aqueous solution.^[^ ^14^, ^15^, ^16^ ^]^

Though crystalline non‐noble transition metal has the advantage in intermediate adsorption free energy, the water dissociation kinetics itself remains sluggish, which is still unsatisfactory for practical alkaline water electrolysis.^[^ ^17^, ^18^ ^]^ Grain boundaries as bulk defects in polycrystalline materials, on the other hand, could stabilize dislocations and generate high‐energy surfaces. This will favor the catalytic kinetics owing to the lattice strain of the catalyst although the active sites in grain boundaries are normally unable to fully exposed.^[^ ^11^, ^19^ ^]^ As a result, the separate drawbacks of polycrystalline and amorphous materials limit their further performance improvement in water electrolysis. Therefore, it is of importance to develop electrocatalysts with judicious configurations that can maintain stability and fully expose the active sites concurrently for further advance of the performance. Previous research has demonstrated that heterostructural transition‐metal based electrocatalysts could favor the catalytic performance. However, most studies have been focused on crystalline grain boundaries and crystalline–crystalline heterostructures, where the character and function of crystalline–amorphous interfaces (CAI) in the catalyst for water electrolysis have scarcely been investigated deeply. It is envisioned that the coexistence of crystalline and amorphous phases in one transition‐metal compound in the form of CAI could be a feasible strategy to accelerate the water electrolysis kinetics.^[^ ^20^, ^21^ ^]^

**Figure 1:**
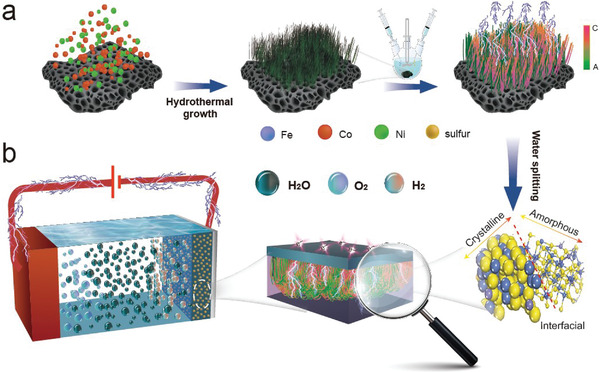
a) Synthesis process for amorphous/crystalline heterostructure FeCo(NiS_2_)_4_‐C/A on Ni foam. b) Schematic illustration of FeCo(NiS_2_)_4_‐C/A as bifunctional electrocatalysts.

Other than interface engineering, the physicochemical properties and electronic structure of the catalyst could be optimized by tuning the components. For example, trimetallic sulfides normally have a lower activation energy than that of monometallic and bimetallic sulfides, which is conducive to electron transfer, thus leading to better electron conductivity.^[^ ^22^, ^23^, ^24^ ^]^ Moreover, the synergistic effects between trimetallic ions allow for the modification of electronic structures, which leads to more active sites and faster reaction kinetics in HER/OER.^[^ ^25^, ^26^, ^27^ ^]^ Therefore, trimetallic sulfides have more applicable features as electrocatalysts and have the potential to outperform monometallic and bimetallic sulfides in terms of electrocatalytic property.^[^ ^2^, ^22^ ^]^ Herein, we report a trimetallic crystalline/amorphous co‐containing FeCo(NiS_2_)_4_ catalyst (denoted as FeCo(NiS_2_)_4_‐C/A), which shows excellent performance toward both HER and OER. This bifunctional FeCo(NiS_2_)_4_‐C/A catalyst was obtained via a series of component transformations as illustrated in **Figure** **1**a. It originates from cobalt‐nickel bimetal (oxy)hydroxides, which were converted into corresponding bimetal sulfides (denoted as CoNiS_x_) and finally underwent a cation‐exchange implementation (details are stated in the Experimental Procedures of the Supporting Information). The as‐obtained FeCo(NiS_2_)_4_‐C/A is composed of both crystalline and amorphous phases, which form abundant CAI. Owing to their unique heterostructure and CAI, the FeCo(NiS_2_)_4_‐C/A exhibits excellent catalytic performances in both HER and OER in alkaline electrolytes (Figure 1b) with fairly low onset overpotentials of 82 and 230 mV, respectively. Density functional theory (DFT) calculations demonstrate that Co atoms on CAI exhibit superior catalytic activity for both HER and OER. Moreover, the bifunctional FeCo(NiS_2_)_4_‐C/A catalyst shows outstanding durability and a low operation voltage of 1.51 V to reach 10 mA cm^−2^ in the two‐electrode configuration for full water splitting.

## 2 Results

### 2.1 Morphology and Electronic Structure Characterizations

**Figure 2:**
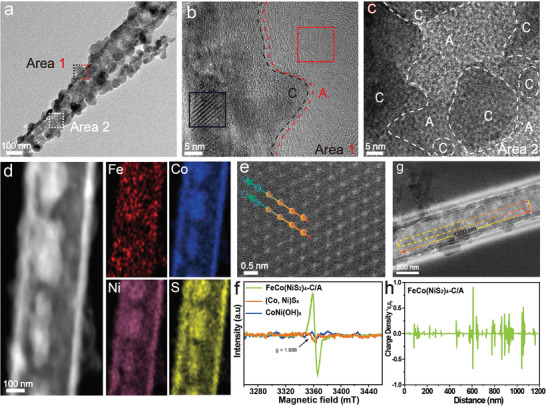
a) TEM of as‐prepared FeCo(NiS_2_)_4_‐C/A. b,c) High‐resolution TEM (HRTEM) image of FeCo(NiS_2_)_4_‐C/A sample. d) HAADF‐mappings and e) spherical aberration‐corrected STEM of FeCo(NiS_2_)_4_‐C/A. f) EPR spectra of CoNi(OH)_x_, CoNiS_x_, and FeCo(NiS_2_)_4_‐C/A. Electron holography of g) FeCo(NiS_2_)_4_‐C/A sample and h) corresponding profiles for charge density distributions.

To confirm the composition and crystal structure of FeCo(NiS_2_)_4_‐C/A, X‐ray powder diffraction (XRD) experiments were conducted. (Figure S1, Supporting Information) exhibits the XRD patterns of FeCo(NiS_2_)_4_‐C/A, CoNiS_x_, and CoNi(OH)_x_. The XRD pattern of CoNi(OH)_x_ showed several peaks with extreme low intensity, indicating it was not well grown via certain crystalline planes. As for CoNiS_x_, few XRD peaks exhibited high intensity, which suggested a poor crystallinity of the material. From scanning electron microscopy (SEM) images in (Figure S2a, Supporting Information), it can be seen that CoNi(OH)_x_ nanoneedle arrays are uniformly grown on Ni foam with a smooth surface at high magnification (Figure S2b, Supporting Information), indicating the well‐prepared bimetallic CoNi(OH)_x_ nanoneedle arrays are ready as a sacrifice template for the next reaction. After the vulcanization process, the needle‐like sample becomes tubular, which is more conducive for CoNiS_x_ to transport ions (Figure S3, Supporting Information). Next, doping of Fe^3+^ into CoNiS_x_ was achieved by an ion exchange process with Fe(NO_3_)_3_ at 80 °C, and it can be observed (Figure S4, Supporting Information) that the formed FeCo(NiS_2_)_4_‐C/A maintains a nanoneedle morphology of the bimetallic CoNiS_x_ precursor while the previous smooth surface of the precursor becomes a rough and nanosheet‐like structure in the final product. Above all, novel heterostructural (crystalline/amorphous) FeCo(NiS_2_)_4_‐C/A were obtained via cation exchange route after incorporating Fe^3+^ into CoNiS_x_, which would favor both HER and OER. FeCo(NiS_2_)_4_‐C/A exhibited tubular structures with a rough surface and the attached flakes, in which dark and light areas can be observed (**Figure** **2**a). The high‐resolution TEM (HRTEM) image of Figure 2b reveals that the FeCo(NiS_2_)_4_‐C/A is composed of crystalline/amorphous mixture, which was also verified by the bright spots (Figure S5a, Supporting Information) and diffused ring (Figure S5b, Supporting Information) in the corresponding selected area of fast Fourier transform (FFT) patterns. Figure 2c confirmed the coexistence of various crystalline and amorphous sections in Area 2, resulting in abundant CAI labeled by dash lines. High‐angle annular dark‐field scanning transmission electron microscopy (HAADF‐TEM) was used to characterize the elemental mapping Fe, Co, Ni, and S that are homogeneously distributed in the hollow structure of FeCo(NiS_2_)_4_‐C/A, and it shows that the material is obtained by cation exchange rather than simple coating (Figure 2d). Spherical aberration‐corrected HAADF‐STEM images revealed that the FeCo(NiS_2_)_4_‐C/A exhibited the typical rhombic phase, where sulfur vacancies sites were observed at the edge of the nanostructure (highlighted as blue dashed circles in Figure 2e). Furthermore, the electron paramagnetic resonance (EPR) spectra of the as‐prepared CoNi(OH)_x_ and CoNiS_x_ did not present any unpaired electrons. In contrast, for FeCo(NiS_2_)_4_‐C/A, a pair of opposite peaks were observed with a signal at *g* = 1.998, suggesting the existence of unpaired electrons due to the sulfur vacancies (Figure 2f).^[^ ^28^, ^29^ ^]^ Off‐axis electron holography was utilized to visualize lattice defects and CAI,^[^ ^30^ ^]^ and the polarization charge density distribution was also quantified (Figure 2g,h). Compared to CoNi(OH)_x_ and CoNiS_x_, FeCo(NiS_2_)_4_‐C/A exhibited an obvious fluctuation of negative and positive charge density, confirming a higher CAI density of FeCo(NiS_2_)_4_‐C/A (Figures S6 and S7, Supporting Information).

Brunauer–Emmett–Teller (BET) surface area and pore diameter of the three samples are characterized by N_2_ sorption isotherm measurements. As plotted in (Figure S8, Supporting Information), the isotherm of FeCo(NiS_2_)_4_‐C/A exhibits a typical H3‐type hysteresis loop in the relative pressure range of 0.3–0.9 P/P_0_ as type IV according to IUPAC protocol.^[^ ^31^ ^]^ This typical curve demonstrates the mesoporous characteristic of FeCo(NiS_2_)_4_‐C/A, and it could be attributed to the porous shell of the hollow‐tube structure. Results calculated out of BET equations indicate the surface area of 662.5 m^2^ g^−1^ and the average pore size obtained from Barrett–Joyner–Halenda (BJH) method of FeCo(NiS_2_)_4_‐C/A show two mesopore sizes of 8.9 and 13.5 nm. The mesopores of FeCo(NiS_2_)_4_‐C/A demonstrate the high surface area and exposure of electrochemically active sites. This structure enables the facile absorption of ions on the surface of electrodes.

**Figure 3:**
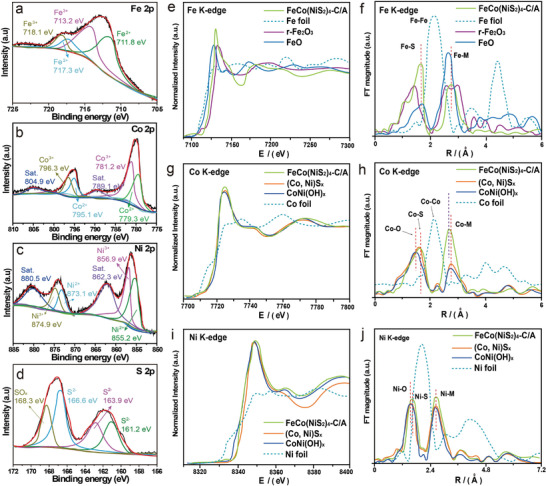
XPS spectra of a) Fe 2p for FeCo(NiS_2_)_4_‐C/A; b) Co 2p for FeCo(NiS_2_)_4_‐C/A; c) Ni 2p and (d) S 2p for FeCo(NiS_2_)_4_‐C/A, respectively. e) Fe K‐edge XANES spectra of FeCo(NiS_2_)_4_‐C/A, and the standard reference Fe foil and FeO powder. g) Co K‐edge XANES spectra of the three samples, and the standard reference Co powder. i) Ni K‐edge XANES spectra of the three samples, and the standard reference Ni foil. Fourier‐transformed EXAFS spectra at the f) Fe K‐edge, h) Co K‐edge, and j) Ni K‐edge collected for FeCo(NiS_2_)_4_‐C/A, (Co,Ni)S_x_, and CoNi(OH)_x_, and the corresponding references.

X‐ray photoelectron spectroscopy (XPS) was applied to investigate the electronic states of metal ions for FeCo(NiS_2_)_4_‐C/A as shown in **Figure** **3**a–d. The Fe 2p spectra (Figure 3a) show the intensity peaks at 718.1/713.2 and 717.3/711.8 eV, indicating the existence of both Fe^3+^ and Fe^2+^ in the compound.^[^ ^32^ ^]^ In Co 2p XPS spectra, Co 2p 3/2 and Co 2p 1/2 are located at 779.3/795.1 and 781.3/796.3 eV, respectively.^[^ ^33^ ^]^ The coexistence of Co^2+^ and Co^3+^ can be revealed from the intensity peaks (Figure 3b). Moreover, for Ni 2p spectra (Figure 3c), two intensity peaks at 855.2/873.1 and 856.9/874.9 eV attributed to Ni 2p 3/2 and Ni 2p 1/2 reveal the valences state of Ni are 2 and 3.^[^ ^34^ ^]^ Therefore, the above results partially prove that a portion of Co^2+^, Ni^2+^, and Fe^2+^ can be oxidized to Co^3+^, Ni^3+^, and Fe^3+^ correspondingly. In Figure 3d, the S 2p XPS peaks at 161.2/163.9/166.6 and 168.3 eV are assigned to S^2−^ and SO_x_ species. It should be noted that the valence state of Fe, Co, and Ni are 2 and 3, which will contribute to the stability of electrode in water splitting in that the valence state could be relatively fixed during the reduction and oxidation circulation on the surface of electrodes.

The X‐ray absorption spectroscopy (XAS) measurements were performed to further investigate the coordination environment and the chemical state of metal species (Fe, Co, and Ni) at the atomic level. The X‐ray absorption near edge structure (XANES) spectra of three samples and corresponding reference at K‐edge are shown in Figure 3e,g,i. The XANES spectra of FeCo(NiS_2_)_4_‐C/A and the reference samples at K‐edge (Figure 3e) reveal that the line position (absorption edge) of FeCo(NiS_2_)_4_‐C/A is located between FeO and Fe_2_O_3_, which indicates that the valence state of Fe species in FeCo(NiS_2_)_4_‐C/A is between Fe^2+^ and Fe^3+^. The Co K‐edge XANES spectra (Figure 3g) show that the Co absorption edge and the green line of FeCo(NiS_2_)_4_‐C/A gradually shifts to the higher‐energy side compared with Co foil, CoNiS_x_, and CoNi(OH)_x_, indicating partial electron transfer from Co to the substitutional Fe or Ni. In the Ni K‐edge XANES spectra (Figure 3i), the binding energy of Ni in FeCo(NiS_2_)_4_‐C/A is a bit higher than that of CoNiS_x_ and CoNi(OH)_x_, implying the average oxidation state of nickel in FeCo(NiS_2_)_4_‐C/A is higher than bivalency. The Fourier transform (FT) *k* ^3^‐weighed extended X‐ray absorption fine structure (EXAFS) spectra of three samples are shown in Figure 3f,h,j. The EXAFS spectrum of FeCo(NiS_2_)_4_‐C/A shows that the main peak is located at 1.79 Å, which is attributed to the Fe—S coordination in the structure (Figure 3f). In addition, Fe—M bonds in FeCo(NiS_2_)_4_‐C/A shift to high‐R with relatively weak peak intensity compared to FeO, caused by the lattice distortion. In the Fourier transformed Co K‐edge EXAFS of the three samples (Figure 3h), CoNi(OH)_x_ presents a dominant peak located at ≈1.52 Å because of the Co—O scattering path. Another deviation peak at ≈1.81 Å is also observed, which matches with the Co—S bond length in FeCo(NiS_2_)_4_‐C/A and CoNiS_x_.^[^ ^35^ ^]^ In addition, the lower intensity of the Co—M (M = Co, Ni, Fe) featured in FeCo(NiS_2_)_4_‐C/A verifies the existence of structural distortion (i.e., disordered atoms and dangling bonds) that reduces the surface energy of FeCo(NiS_2_)_4_‐C/A and improves the structural durability of the catalyst.^[^ ^2^, ^15^ ^]^ In the Fourier transform of the Ni K‐edge EXAFS spectra of FeCo(NiS_2_)_4_‐C/A, CoNiS_x_, and CoNi(OH)_x_ (Figure 3j), both Fe—S and Fe—M bonds in FeCo(NiS_2_)_4_‐C/A shift to high‐R with relatively high peak intensity, which is attributed to the valence state of nickel (>2^+^). After the introduction of iron, the peak of Ni‐M in FeCo(NiS_2_)_4_ did not shift compared to two control groups. The peak of Co—M in FeCo(NiS_2_)_4_ shifted to left by 0.1 Å, indicating the ligancy of Co and other metals increased. At the same time, the peak of Co—S in FeCo(NiS_2_)_4_ shifted to right by 0.05 Å, suggesting the ligancy of Co and S decreased. Therefore, the coordination between Co and S changed after the introduction of Fe, more Co atoms coordinated with Ni and Fe atoms rather than S atoms, thus contributing to the generation of sulfur vacancies. The above results demonstrate that the cation‐exchange process led to the formation of defective lattice and coordinatively unsaturated metal centers, which will function as the active sites in OER.^[^ ^2^ ^]^

### 2.2 DFT Calculations of Crystalline/Amorphous Interface

In this work, the DFT and ab initio molecular dynamics (AIMD) simulations are coupled to reveal the high‐performance water splitting mechanism of FeCo(NiS_2_)_4_‐C/A. Previously reported research lacks theoretical study on the function of CAI in water splitting process, where the completed and accurate construction of CAI model is the key. Herein, we employ the AIMD simulation to establish the FeCo(NiS_2_)_4_‐C/A. Experimental results found abundant sulfur vacancies in FeCo(NiS_2_)_4_‐C/A, which may explain the formation of amorphous structure. Thus, the sulfur vacancies were created in part I to build the amorphous counterpart near CAI in FeCo(NiS_2_)_4_‐C/A, shown in (Figure S9a, Supporting Information). After 10 ps MD simulations, the temperature and potential energy tend to be stable (Figure S9b, Supporting Information), confirming the successful formation of amorphous/crystalline heterostructure in FeCo(NiS_2_)_4_‐C/A (Figure S9c, Supporting Information). H_2_O molecules adsorption behavior regarding different counterparts of FeCo(NiS_2_)_4_‐C/A are analyzed to investigate the catalytic kinetics of water splitting. H_2_O molecules adsorbed on Ni atoms on the crystalline surface of FeCo(NiS_2_)_4_‐C/A (see the position of a1) exhibit an adsorption energy of −0.07 eV (Figure S9d, Supporting Information). At the CAI of FeCo(NiS_2_)_4_‐C/A, the H_2_O molecule adsorbed on the sulfur atom shows a lower adsorption energy of −0.18 eV, decreased by 0.11 eV (Figure S9e, Supporting Information). The adsorption energy continues to decrease when it comes to the adsorption between H_2_O molecules and interfacial Ni atoms (−0.26 eV, Figure S8f, Supporting Information). The adsorption energy then increases to −0.12 eV on Ni atoms on the amorphous surface (Figure S9g, Supporting Information). The above theoretical study indicates that H_2_O molecules are inclined to be adsorbed on the CAI of FeCo(NiS_2_)_4_‐C/A, especially on the interfacial Ni atoms. To study H atoms adsorption configurations of FeCo(NiS_2_)_4_‐C/A heterostructure, different adsorption sites from crystalline to amorphous counterparts are taken into account. For instance, H atom adsorbed on the Ni atom at position e1 has an adsorption energy of 0.53 eV (Figure S8h, Supporting Information), suggesting a weak interaction between the H atom and crystalline counterparts of FeCo(NiS_2_)_4_. Co atoms at CAI exhibit a low H‐atom adsorption energy of 0.002 eV, favoring both H_2_ capture and release (Figure S9i, Supporting Information). When it comes to the adsorption between H atoms and interfacial Ni atoms, the adsorption energy of 0.72 eV (Figure S9j, Supporting Information) indicates H atoms are less prone to interact with interfacial Ni atoms. The S atom on the surface of amorphous counterparts of FeCo(NiS_2_)_4_‐C/A has an adsorption energy of 0.2 eV toward the H atom (Figure S9k, Supporting Information). This transition of the H‐atom adsorption energy from crystalline to amorphous counterparts through the interface regarding different host atoms demonstrates that Co atoms at CAI will contribute to the better catalytic performance of HER.

**Figure 4:**
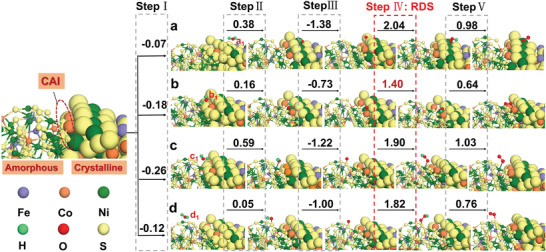
Oxygen evolution reaction (OER) processes on the amorphous/crystalline FeCo(NiS_2_)_4_‐C/A heterostructure at a) a_1_, b) b_1_, c) c_1_, and d) d_1_ positions, respectively.

The exploration of OER catalytic pathways in terms of different counterparts from the crystalline through CAI to the amorphous (position a_1_, b_1_, c_1_, and d_1_) are displayed in **Figure** **4**. Step I shows the H_2_O molecule adsorption energy. Steps II and III depict the dehydrogenation process of forming *OH and *O species, respectively. Steps IV and V describe the required energy to form *OOH intermediate and O_2_. For Step IV, it has a higher reaction energy barrier over other steps in the pathway, defined as the rate‐determining step (RDS), which is the key step to perform the OER catalytic process. Position b_1_ retains a remarkably lower energy barrier of 1.40 eV in the RDS than other selected positions (Figure 4b). For step I and II, position b_1_ at CAI exhibits an excellent activity over the H_2_O molecule adsorption and *OH formation, very close to position c_1_ and d_1_ that possess the best performance of step I and II, respectively (Figure 4c,d). For step III, it is an exothermal reaction, thus this step will not be the barrier of the whole OER pathway. Furthermore, *OOH catalyzed on the position b1 to generate O_2_ at step V requires the lowest energy of 0.64 eV in contrast with other catalytic positions. Overall, position b_1_ exhibits the leading OER catalytic activity over other selected positions based on the above theoretical study, verifying that CAI will promote the OER process. It is noteworthy that Co atoms at CAI will avail the catalytic activity of OER.

### 2.3 Electrochemical Water Splitting

**Figure 5:**
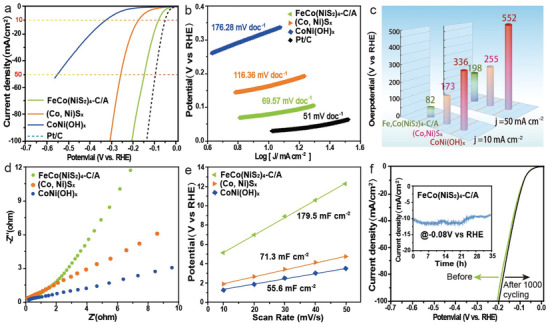
HER performances of as‐synthesis catalysis. a) LSV curves of different electrocatalysts in 1.0 m KOH solution. b) Corresponding HER Tafel plots. c) Overpotential histogram of LSV. d) Nyquist plots and e) ECSA estimated by *C*_dl_ values. f) LSV polarization curves of the CoFe(NiS_2_)_4_‐C/A catalyst after 1000 cycles with current density versus time (*i*–*t*) curves of HER for over 35 h shown in the inset.

These conversions based on the oxidation and reduction of triple metal ions will increase the electrocatalytic capacity compared with CoNiS_x_ and CoNi(OH)_x._ The electrocatalytic OER and HER activity were studied via the linear sweep voltammetry (LSV). The electrocatalytic activity of FeCo(NiS_2_)_4_‐C/A toward HER was investigated under alkaline electrolyte (1 m KOH) using a three‐electrode system (Experimental Section). Polarization curves were measured by LSV with a scan rate of 5 mV s^−1^ under *iR*‐correction. In addition, all potentials were converted to those at a reversible hydrogen electrode (RHE) scale. FeCo(NiS_2_)_4_‐C/A showed comparable electrocatalytic performance to Pt/C (**Figure** **5**). Tafel slope can be used as an indicator of HER and OER activity of catalysts as the smaller Tafel slope represents the faster chemical reaction kinetics. Figure 5b shows the Tafel slope for all samples FeCo(NiS_2_)_4_‐C/A (69.57 mV dec^−1^), CoNiS_x_ (116.36 mV dec^−1^), CoNi(OH)_x_ (176.28 mV dec^−1^), and Pt/C (51 mV dec^−1^). The lower Tafel slope of FeCo(NiS_2_)_4_‐C/A indicates a faster chemical reaction kinetic of HER.^[^ ^36^ ^]^ Meanwhile, FeCo(NiS_2_)_4_ only needs an overpotential of 82 mV to reach the current density of −10 mA cm^−2^, which is 91 and 254 mV lower than that of CoNiS_x_ and CoNi(OH)_x_, respectively (Figure 5c). Furthermore, the current density of FeCo(NiS_2_)_4_‐C/A can be scaled up to 50 mA cm^−2^ at an overpotential of 198 mV, which can be compared favorably to those reported metal chalcogenides and other earth‐abundant electrocatalysts (Table S1, Supporting Information). In addition, the reaction kinetics of the as‐synthesized samples were investigated by electrochemical impedance spectroscopy (EIS), shown in Figure 5d. It is clear that FeCo(NiS_2_)_4_‐C/A has the smallest *R*_ct_ value among all samples, implying that the energy barrier of electrode transfer was reduced at the electrode–electrolyte interface, thus leading to the enhanced reaction kinetics. The ratio of the electrochemical active surface (ECSA) to the two‐layer capacitance (*C*_dl_) can reflect the electrocatalytic activity.^[^ ^37^ ^]^ To measure the electrochemical property, the ECSA of sample FeCo(NiS_2_)_4_‐C/A was further analyzed by cyclic voltammetry (CV) at 1.0 m KOH (Figure S10, Supporting Information). It is revealed by Figure 5e that the *C*_dl_ value of the FeCo(NiS_2_)_4_‐C/A is the top (179.5 mF cm^−2^) among all samples. This result indicates that FeCo(NiS_2_)_4_‐C/A has more reaction surface active sites to be the ideal electrocatalyst corresponding to the LSV results. The durability of the sample of FeCo(NiS_2_)_4_‐C/A in the KOH electrolyte was detected by 1000 scanning cycles of the electrocatalyst (Figure 5f). Cyclic stability of FeCo(NiS_2_)_4_‐C/A was evaluated by chronopotentiometry at 0.08V versus RHE (Figure 5f, inset). After continuous electrolysis for 35 h, the current density of the FeCo(NiS_2_)_4_‐C/A remained with a minor fluctuation, verifying the outstanding HER‐stability of FeCo(NiS_2_)_4_‐C/A in the strongly alkaline medium, significantly improved compared to CoNiS_x_ and CoNi(OH)_x_ (Figure S11, Supporting Information). In addition, Fe_0.5_Co(NiS_2_)_4_‐C/A and Fe_1.5_Co(NiS_2_)_4_‐C/A were synthesized at different amounts of Fe introduced into the compounds and their catalytic performances were evaluated (Figure S12, Supporting Information). The Holo experiment results revealed that Fe_0.5_Co(NiS_2_)_4_‐C/A and Fe_1.5_Co(NiS_2_)_4_‐C/A did exhibit amorphous/crystalline interfaces (Figure S12a–d, Supporting Information). Moreover, Fe_0.5_Co(NiS_2_)_4_‐C/A and Fe_1.5_Co(NiS_2_)_4_‐C/A exhibited poor catalytic performance compared to FeCo(NiS_2_)_4_‐C/A. Therefore, the above results indicated that FeCo(NiS_2_)_4_‐C/A possesses a superior nanostructure with an appropriate amount of Fe, leading to the best performance in terms of both HER and OER.

**Figure 6:**
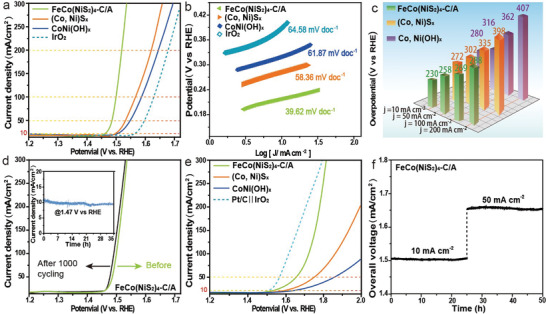
OER performances of as‐synthesis catalysis. a) LSV curves of different electrocatalysts in 1.0 m KOH solution. b) Corresponding HER Tafel slopes. c) Overpotential histogram of LSV. d) LSV polarization curves of the CoFe(NiS_2_)_4_‐C/A catalyst after 1000 cycles with current density versus time (*i*–*t*) curves of OER for over 35 h shown in the inset. e) CoFe(NiS_2_)_4_‐C/A for overall water splitting electrocatalysis in 1 m KOH in two‐electrode system. f) Long‐term stability tests carried out at 10 and 50 mA cm^−2^ for over 50 h.

The electrocatalytic ability of the FeCo(NiS_2_)_4_‐C/A for OER was measured under the same condition as that of HER. The OER performance of CoNiS_x_, CoNi(OH)_x_, and IrO_2_ on carbon sheet were also tested for comparison. In contrast with IrO_2,_ CoNiS_x_, and CoNi(OH)_x_, FeCo(NiS_2_)_4_‐C/A showed a low onset potential of ≈1.46 V (**Figure** **6**a,c). At a lower overpotential of 230 mV, the current density of FeCo(NiS_2_)_4_ reached 10 mA cm^−2^ and further increased to 200 mA cm^−2^ rapidly with the lowest overpotential of 292 mV, which is comparable to the reported best OER catalyst. The capacity of FeCo(NiS_2_)_4_‐C/A regarding overpotentials is comparable to those of recently reported earth‐abundant OER catalysts with high performance (Table S2, Supporting Information). The catalytic kinetics of the presented catalysts was further evaluated by the Tafel slopes (Figure 6b). As expected, FeCo(NiS_2_)_4_‐C/A possesses a much smaller Tafel slope of 39.62 mV dec^−1^ than that of CoNiS_x_, CoNi(OH)_x_, and IrO_2_, suggesting its distinct OER kinetics. The LSV curves (Figure 6d) remained almost unchanged after 1000 cycles, with an error of less than 1%, proving the excellent durability of the FeCo(NiS_2_)_4_‐C/A. OER stability of FeCo(NiS_2_)_4_‐C/A was tested by chronopotentiometry at 0.08V vs RHE in 1 m KOH solution. FeCo(NiS_2_)_4_‐C/A exhibited long‐term stability as the current density remained stable for 35 h (the inset part in Figure 6d). CoNiS_x_ and CoNi(OH)_x_ exhibited lower stability and higher overpotential under the current density of 10 mA cm^−2^ (Figure S13, Supporting Information), further confirming that the stability and OER catalytic performance have been optimized by FeCo(NiS_2_)_4_‐C/A. The OER catalytic performance of FeCo(NiS_2_)_4_‐C/A is mainly attributed to the high surface area generated by the outer film of nanotubes, the exposed active sites resulting from amorphous structure, and the optimized electroconductivity and charge transfer anchored in the CAI between amorphous and crystalline counterparts. To further verify the stability of FeCo(NiS_2_)_4_‐C/A, the testing condition of 50 mA cm^−2^ for 35 h was applied. It is found that FeCo(NiS_2_)_4_‐C/A maintained its stability after the 35‐h IT test (Figure S14, Supporting Information). Moreover, CAI of FeCo(NiS_2_)_4_‐C/A was not transformed or destructed after the stability test, displayed in Figure S15 (Supporting Information).

In order to investigate the effect of CAI on the catalytic performance of water splitting, FeCo(NiS_2_)_4_ with different composition of crystalline and amorphous counterparts were fabricated (denoted as FeCo(NiS_2_)_4_‐A^+^, FeCo(NiS_2_)_4_‐C^+^, and FeCo(NiS_2_)_4_‐C^++^). The XRD pattern revealed the crystalline structure and of the above mentioned three samples in Figure S16 (Supporting Information). ECSA results demonstrated the electrochemical active area of FeCo(NiS_2_)_4_‐C^+^ and FeCo(NiS_2_)_4_‐C^++^ in Figure S17 (Supporting Information). Heterostructural FeCo(NiS_2_)_4_ containing more amorphous counterparts and less crystalline counterparts (denoted as FeCo(NiS_2_)_4_‐A^+^) was fabricated via tuning the reaction condition (Materials Synthesis, Supporting Information) to study the function and mechanism of CAI in water splitting performance. Thin films affiliated to the tubal structure can be seen from (Figure S18a, Supporting Information), exhibiting an amorphous characteristic in the HRTEM image (Figure S18b, Supporting Information). Figure S18c (Supporting Information) shows the boundary of the thin film and hollow tube while the amorphous and crystalline counterparts are clearly revealed by areas A and C in the HRTEM image. Crystalline structure with different crystallinity inside the hollow tube is characterized through divided area C in (Figure S18d, Supporting Information). According to Figure S18e,f (Supporting Information), HER performance of FeCo(NiS_2_)_4_‐C/A is superior to FeCo(NiS_2_)_4_‐A^+^. FeCo(NiS_2_)_4_‐C/A showed a lower onset potential of −0.2 V and Tafel slope of 69.57 mV doc^−1^ compared to FeCo(NiS_2_)_4_‐A^+^. For OER, FeCo(NiS_2_)_4_‐A^+^ exhibited similar performance to FeCo(NiS_2_)_4_‐C/A, and its Tafel slope is 42.25 mV doc^−1^, which is very close to that of FeCo(NiS_2_)_4_‐C/A (39.62 mV doc^−1^) (Figure S12g,h, Supporting Information). It can be concluded that the amorphous counterpart in FeCo(NiS_2_)_4_ A^+^ may contribute to its OER performance. Nevertheless, FeCo(NiS_2_)_4_‐C/A possesses outstanding catalytic activity in both HER and OER due to more CAI components.

Heterostructural FeCo(NiS_2_)_4_ containing more crystalline counterparts and less amorphous counterparts (FeCo(NiS_2_)_4_‐C^+^) was synthesized by altering the synthesis methodology (Materials Synthesis, Supporting Information) to further demonstrate the importance of CAI in water splitting catalysts. Flake‐like structure in the outer part of FeCo(NiS_2_)_4_‐C^+^ hollow tube (Figure S19a, Supporting Information) is characterized by HRTEM. Crystalline structure dominates in the FeCo(NiS_2_)_4_‐C^+^ hollow tube, marked as area C in the HRTEM image (Figure S19b, Supporting Information). The HER performance of FeCo(NiS_2_)_4_‐C^+^ is close to FeCo(NiS_2_)_4_‐C/A (Figure S19c, Supporting Information). Tafel slope of FeCo(NiS_2_)_4_‐C^+^ is 78.25 mV doc^−1^, a bit higher than that of FeCo(NiS_2_)_4_ C/A (69.57 mV doc^−1^, Figure S13d, Supporting Information). Overall, the HER catalytic activity of FeCo(NiS_2_)_4_‐C^+^ is comparable to FeCo(NiS_2_)_4_‐C/A. For OER catalysis, FeCo(NiS_2_)_4_‐C^+^ exhibited a moderate activity compared to FeCo(NiS_2_)_4_‐C/A (Figure S13e, Supporting Information). Tafel slope (Figure S19f, Supporting Information) reveals the difference of OER performance between FeCo(NiS_2_)_4_‐C/A (39.62 mV doc^−1^) and FeCo(NiS_2_)_4_‐C^+^ (60.16 mV doc^−1^), indicating the crystalline counterpart is more favorable to HER performance. FeCo(NiS_2_)_4_‐C/A still outperforms FeCo(NiS_2_)_4_‐C^+^ in both HER and OER owing to the existence of CAI.

To further demonstrate the function of CAI in water splitting catalysts, the comparison of grain boundaries and CAI regarding HER and OER catalytic performance is addressed. FeCo(NiS_2_)_4_‐C^++^ rich in grain boundaries (Figure S20a, Supporting Information) that generated from anisotropic crystalline counterparts is obtained via a multistep synthetic route (Materials Synthesis, Supporting Information). Anisotropic crystalline counterparts are segmented and marked as area C, and grain boundaries are labeled as dot lines on the edge of different area C, characterized by HRTEM (Figure S20b, Supporting Information). FeCo(NiS_2_)_4_ C^++^ rich in grain boundaries showed moderate catalytic activity in HER and OER compared to FeCo(NiS_2_)_4_‐C/A with abundant CAI (Figure S20c–f, Supporting Information). Therefore, based on the above discussion of computational study and experimental results, FeCo(NiS_2_)_4_‐C/A with abundant CAI surpasses FeCo(NiS_2_)_4_‐A^+^, FeCo(NiS_2_)_4_‐C^+^, and FeCo(NiS_2_)_4_‐C^++^ in terms of both HER and OER catalysis. The existence of CAI in FeCo(NiS_2_)_4_‐C/A is the key factor in improving water splitting efficiency. The significance of CAI in optimizing the bifunctional water splitting catalysts is conclusive. The stability of FeCo(NiS_2_)_4_‐C/A, FeCo(NiS_2_)_4_‐C^+^, and FeCo(NiS_2_)_4_‐C^++^ was investigated at a current density of 10 mA cm^−2^ for 75 h. All samples maintained its stability before 40 h. Minor difference was observed after comparing all samples with different compositions of crystalline counterparts from 40 to 75 h (Figure S21, Supporting Information), indicating great stability FeCo(NiS_2_)_4_‐C/A at a current density of 10 mA cm^−2^. The stability of FeCo(NiS_2_)_4_‐C/A, FeCo(NiS_2_)_4_‐C^+^, and FeCo(NiS_2_)_4_‐C^++^ was also studied at a current density of 50 mA cm^−2^ for 75 h. FeCo(NiS_2_)_4_‐C/A maintained its stability before 40 h. FeCo(NiS_2_)_4_‐C^+^ and FeCo(NiS_2_)_4_‐C^++^ with more crystalline counterparts exhibited better stability for 75 h (Figure S22, Supporting Information). The stability results confirmed that a high percentage of crystalline in the nanomaterial favors long‐term stability of the catalyst, and the stability of FeCo(NiS_2_)_4_‐C/A at a higher current density is stronger than that at a lower current density.^[^ ^16^, ^38^ ^]^

Single FeCo(NiS_2_)_4_‐C/A electrode bifunctionally catalyzing both HER and OER could greatly simplify the water splitting system and reduce the production cost. Encouraged by the impressive catalytic activity of the FeCo(NiS_2_)_4_‐C/A in terms of both HER and OER, we then assembled a water electrolyzer employing the FeCo(NiS_2_)_4_‐C/A as anode and cathode for overall water splitting test in 1 M KOH. The CoNiS_x_||CoNiS_x_, CoNi(OH)_x_||CoNi(OH)_x_, and Pt/C||IrO_2_ were all loaded on the carbon sheet to be compared as control groups. As shown in Figure 6e, water splitting catalyzed by CoNi(OH)_x_ and CoNiS_x_ requires cell voltages of 1.65 and 1.59 V to drive 10 mA cm^−2^, respectively. In contrast, the cell voltage demanded for FeCo(NiS_2_)_4_‐C/A is as low as 1.51 V, quite close to that of Pt/C||IrO_2_ (1.5 V). It is noteworthy that FeCo(NiS_2_)_4_‐C/A electrodes can drive high current densities (e.g., 300 mA cm^−2^) at cell voltages that are comparable to those of Pt/C||IrO_2_, which is beneficial to practical H_2_ production, making itself a competitive electrocatalyst for water splitting (Table S3, Supporting Information). In addition, FeCo(NiS_2_)_4_‐C/A also showed potent durability, tested by the chronopotentiometry for over 50 h (Figure 6f). The cell voltage of FeCo(NiS_2_)_4_‐C/A regarding HER/OER remained stable for 50 h. The current density was increased from 10 to 50 mA cm^−2^ after 25 h of operation. The faradaic efficiency of FeCo(NiS_2_)_4_ was calculated to be ≈97% by comparing the experimental H_2_/O_2_ production with theoretical values (Figure S23, Supporting Information).

## 3 Conclusion

In summary, FeCo(NiS_2_)_4_‐C/A with abundant CAI that possesses an outstanding catalytic performance of water electrolysis has been constructed via a cation‐exchange route. The incorporation of Fe ions not only tunes the amorphous/crystalline heterogeneity that induces rich surface defects, the exposure of active sites, and a higher density of CAI, but also alters the morphology of FeCo(NiS_2_)_4_‐C/A to hollow nanotubes with porous structure, favoring the ion exchange and charge transfer in water splitting. Consequently, the heterostructural FeCo(NiS_2_)_4_‐C/A presents low overpotentials, high current densities, and prominent stability for both HER and OER. The overall current density of 10 mA cm^−2^ can be achieved at a low cell voltage of 1.51 V using FeCo(NiS_2_)_4_‐C/A as electrodes in 1 m KOH. Based on rigorous experimental and computational studies, the superiority and function of CAI in bifunctional FeCo(NiS_2_)_4_‐C/A electrocatalysts have been demonstrated, explaining the kinetics behind the excellent performance of FeCo(NiS_2_)_4_‐C/A in water electrolysis. The presented work pointed out the value of CAI of electrocatalysts, which may give a direction on the path of designing the ideal electrocatalysts for water splitting.

## Conflict of Interest

The authors declare no conflict of interest.

## Supporting information

Supporting Information

## Data Availability

The data that support the findings of this study are available from the corresponding author upon reasonable request.
