# Supplementary material for: Unveiling the Accelerated Water Electrolysis Kinetics of Heterostructural Iron‐Cobalt‐Nickel Sulfides by Probing into Crystalline/Amorphous Interfaces in Stepwise Catalytic Reactions
Source: Adv Sci (Weinh). 2022 Sep 4;9(30):2201903. doi: 10.1002/advs.202201903 (PMC9596816; doi:10.1002/advs.202201903)
Supplement: Supplementary file 1 — Supporting Information [file ADVS-9-2201903-s001.pdf]

Supporting Information

Unveiling the Accelerated Water Electrolysis Kinetics of Heterostructural Iron-Cobalt-Nickel Sulfides by Probing into Crystalline/Amorphous Interfaces in Stepwise Catalytic Reactions

Zhengxiang Gu, Yechuan Zhang, Xuelian Wei, Zhenyu Duan, Long Ren, Jiecheng Ji, Xiaoqin Zhang, Yuxin Zhang, Qiyong Gong, Hao Wu, Kui Luo,**

Dr. Z. Gu, Y. Zhang, Dr. Z. Duan, Dr. L. Ren, Dr. J. Ji, Dr. X. Zhang, Y. Zhang, Prof. Q. Gong, Prof. K. Luo
Huaxi MR Research Center (HMRRC), Animal Experimental Center, Department of Radiology, National Clinical Research Center for Geriatrics, Frontiers Science Center for Disease-Related Molecular Network, State Key Laboratory of Biotherapy, West China Hospital, Sichuan University, Chengdu, 610041, China.
*E-mail: luokui@scu.edu.cn

Y. Zhang
School of Chemical Engineering and Advanced Materials, University of Adelaide, SA 5005, Australia.

X. Wei
National Engineering Research Center for Biomaterials, Sichuan University, 29 Wangjiang Road, Chengdu 610064, China

Prof. Q. Gong, Prof. K. Luo
Functional and Molecular Imaging Key Laboratory of Sichuan Province, and Research Unit of Psychoradiology, Chinese Academy of Medical Sciences, Chengdu, 610041, China

Prof. H. Wu
Institute of Molecular Sciences and Engineering, Institute of Frontier and Interdisciplinary Science, Shandong University, Qingdao, Shandong, 266237, China. *E-mail: haowu2020@sdu.edu.cn

*Corresponding authors.

Content

Supplementary Text 3

1. Experimental section 3

1. 1 Material synthesis 3
1.1.1 Synthesis of CoNi(OH)$_x$ 3
1.1.2 Synthesis of CoNiS$_x$ 3
1.1.3 Synthesis of FeCo(NiS$_2$)$_4$-C/A 3
1.1.4 Synthesis of FeCo(NiS$_2$)$_4$-A$^+$ 3
1.1.5 Synthesis of FeCo(NiS$_2$)$_4$-C$^+$ 4
1.1.6 Synthesis of FeCo(NiS$_2$)$_4$-C^{++} 4
1. 2 Structure characterizations 4
1. 3 Calculation methodology 4
1. 4 Electrochemical measurements 5
1. 5 Overall water splitting electrocatalysis 6
1. 6 Faradaic efficiency measurements 6
2. Supplementary Results 7

2.1 Supplementary Figures 7
2.2 Supplementary tables 21

Supplementary Text

1. Experimental section

1. 1 Material synthesis

1.1.1 Synthesis of CoNi(OH)$_x$

1 mmol $CoCl_2 \cdot 6H_2O$, 1 mmol $NiCl_2 \cdot 6H_2O$, 4 mmol NH_4F, and 10 mmol urea were mixed in 50 ml deionized water with a 1.5 X 2.5 cm Ni foam, and stirred for 30 min. The previous mixture was transferred to a 100 ml pressure vessel and reacted at 100 °C for 10 h. NiCo(OH)$_x$ on Ni foam was washed with water and ethanol for 3 times.[S1,S2]

1.1.2 Synthesis of CoNiS$_x$

Ni foam with NiCo(OH)$_x$ were immersed in 45ml deionized water in a 100 ml three-neck flask. 0.5 ml 20-24 wt% $(NH_4)_2S$ aqueous solution was then injected to the three-neck flask at a speed of 50 μL min^{-1} via a micro injection pump under the N_2 protection. The mixture was heated to 60 °C at 2 °C min^{-1} under N_2 protection and held for 60 min. CoNiS$_x$ was washed off from Ni foam with deionized water after the previous solution was cooled down to room temperature. CoNiS$_x$ was washed with ethanol and water for three times, collected by centrifugation, and dried at 60 °C for 12 h.

1.1.3 Synthesis of FeCo(NiS$_2$)$_4$-C/A

Ni foam with NiCo(OH)$_x$ were immersed in 45ml deionized water in a 100 ml three-neck flask. 0.5 ml 20-24 wt% $(NH_4)_2S$ aqueous solution was then injected to the three-neck flask at a speed of 50 μL min^{-1} via a micro injection pump under the N_2 protection. Afterwards, 10 mL of 1mmol $FeCl_3 \cdot 4H_2O$ was added to the previous solution at a speed of 100 μL min^{-1} using the injection pump. The mixture was heated to 80 °C at 2 °C min^{-1} under N_2 protection and held for 120 min. Ni foam with the mixture were transfer to a steel reactor and heated at 100 °C for 2 h. FeCo(NiS$_2$)$_4$-C/A was washed with ethanol and water for three times, collected by centrifugation, and dried at 60 °C for 12 h.

1.1.4 Synthesis of FeCo(NiS$_2$)$_4$-A$^+$

Ni foam with NiCo(OH)$_x$ were immersed in 45ml deionized water in a 100 ml three-neck flask. 0.5 ml 20-24 wt% $(NH_4)_2S$ aqueous solution was then injected to the three-neck flask at a speed of 50 μL min^{-1} via a micro injection pump under the N_2 protection. Afterwards, 10 mL of 1mmol $FeCl_3 \cdot 4H_2O$ was added to the previous solution at a speed of 100 μL min^{-1} using the injection pump. The mixture was heated to 80 °C at 2 °C min^{-1} under N_2 protection and held for 8 h. Ni foam with the mixture were transfer to a steel reactor and heated at 80 °C for

20 h. $FeCo(NiS_2)_4$-A^+ was washed with ethanol and water for three times, collected by centrifugation, and dried at 60 °C for 12 h.

1.1.5 Synthesis of $FeCo(NiS_2)_4$-C^+

Ni foam with $NiCo(OH)_x$ were immersed in 45ml deionized water in a 100 ml three-neck flask. 0.5 ml 20-24 wt% $(NH_4)_2S$ aqueous solution was then injected to the three-neck flask at a speed of 50 μL min^{-1} via a micro injection pump under the N_2 protection. Afterwards, 10 mL of 1mmol $FeCl_3\cdot 4H_2O$ was added to the previous solution at a speed of 100 μL min^{-1} using the injection pump. The mixture was heated to 80 °C at 2 °C min^{-1} under N_2 protection and held for 2 h. Ni foam with the mixture were transfer to a tube furnace with N_2 condition and heated at 180 °C for 3 h. $FeCo(NiS_2)_4$-C^+ was washed with ethanol and water for three times, collected by centrifugation, and dried at 60 °C for 12 h.

1.1.6 Synthesis of $FeCo(NiS_2)_4$-C^{++}

Ni foam with $NiCo(OH)_x$ were immersed in 45ml deionized water in a 100 ml three-neck flask. 0.5 ml 20-24 wt% $(NH_4)_2S$ aqueous solution was then injected to the three-neck flask at a speed of 50 μL min^{-1} via a micro injection pump under the N_2 protection. Afterwards, 10 mL of 1mmol $FeCl_3\cdot 4H_2O$ was added to the previous solution at a speed of 100 μL min^{-1} using the injection pump. The mixture was heated to 80 °C at 2 °C min^{-1} under N_2 protection and held for 2 h. Ni foam with the mixture were transfer to a tube furnace with N_2 condition and heated at 300 °C for 3 h. $FeCo(NiS_2)_4$-C^{++} was washed with ethanol and water for three times, collected by centrifugation, and dried at 60 °C for 12 h.

1. 2 Structure characterizations

A field emission scanning electron microscopy (FESEM; ZEISS-Merlin), a transmission electron microscopy (TEM, JEOL-2010) with energy dispersive X-ray spectroscopy (EDX), and a high-resolution TEM (HRTEM, JEOL-2010) are used to characterize the morphology and composition of samples. (XRD) curves of samples were recorded on Rigaku at 40 kV and 40 mA, and X-ray photoelectron spectroscopy (XPS) curves are obtained on a PHI Quantera SXM (ULVAC-PHI) instrument to determine the compositions and the valence states of the elements in the samples.

1. 3 Calculation methodology

In this work, the AIMD calculations are performed by CP2K/Quickstep.[S3] The Perdew-Burke-Ernzerhof (PBE) function is used. The Geodecker-Teter-Hutter (GTH) pseudopotentials are adopted.[S4] The plane wave cutoff is set to 350 Ry, and the Gaussian basis set uses double-ζ with one set of polarization function (DZVP).[S5] The canonical

ensemble (NVT) conditions are imposed by a Nose-Hoover thermostat, and the temperature is set to 300 K. The time step is set to 2 fs, and the whole simulation time is more than 15 ps.

To analyze the water splitting reaction pathways, the Vienna ab initio Simulation Package (VASP) at the generalized gradient approximation (GGA)/Perdew-Burke-Ernzerhof (PBE) level is applied.[S6,S7] The gamma type of k-point sample is used. The plane wave cutoff is set to 400 eV in the projector augmented wave (PAW) potentials. A Gaussian smearing of 0.05 eV is used to calculate the self-consistence and convergence of electron density. All atomic positions are allowed to relax until the forces are less than 0.02 eV/Å. The electron convergence energy is set as 10^{-5} eV. A vacuum of at least 15 Å is used in these monolayer models to avoid the interactions between its periodic images. The DFT-D3 scheme is adopted to correct the van der Waals interaction.[S8]

To calculate the reaction free energy profile of the oxygen evolution reaction (OER), the computational hydrogen electrode (CHE) model is adopted.[S9] The reaction free energies is defined as:

$$\Delta G = \Delta E + \Delta ZPE - T\Delta S \quad (1)$$

where ΔE, ΔZPE and ΔS stand for the reaction energy, differences in zero-point energy and entropy, respectively.

1. 4 Electrochemical measurements

All electrochemical measurements were performed on a CHI 760E electrochemical work station with a typical three-electrode setting at room temperature. A graphite rod and a Hg/HgO electrode were selected as a counter and reference electrode, respectively. The self-supporting array grown on carbon cloth (1×1 cm, mass loading ~ 2.5 mg/cm^{-2}) was directly used as a working electrode. The electrochemical data were collected in an electrolyte of 1.0 M KOH. The measured potentials via the Hg/HgO electrode were converted to those based on a reversible hydrogen electrode (RHE) by the Nernst equation: E(RHE) = E(Hg/HgO) + 0.0591*pH + 0.098. The overpotential (η) was calculated according to the following equation: η = E(RHE)-1.23 V. Linear sweep voltammetry curves for OER and HER were established at a scanning rate of 5 mV/s before 50 cycles of the cyclic voltammetry tests at a scan rate of 50 mV/s to obtain stable curves. The Tafel slopes were obtained from the polarization curves by the equation, η = a + b log (i). The chronoamperometry was operated to evaluate the stability under different current densities. Electrochemical impedance spectroscopy (EIS) measurements were performed over a frequency range of 0.1-10^6 Hz by applying an AC amplitude of 50 mV. All data presented were within 90% iR-correction.

1. 5 Overall water splitting electrocatalysis

The $FeCo(NiS_2)_4$-C/A loaded on carbon sheet was used as both the positive and negative electrodes. The electrocatalytic activity of the working electrode towards the overall water splitting was examined by polarization curves using linear sweep voltammetry (LSV) in 1.0 M KOH at a scan rate of 5 mV s^{-1}. The long-term durability test was performed using chronoamperometric measurements. All data presented were within 90% iR-correction.

1. 6 Faradaic efficiency measurements

The quantitative measurement of O_2 and H_2 was carried out using displacement method. The water splitting experiment was performed in an airtight diaphragm cell. The $(Co, Ni, Fe)_9S_8$ loaded on Ni foam was used as both the positive and negative electrodes, which were inserted into each side of the cell. The volume of each side of the cell is about 25 mL. Prior to the test, each side of the cell was filled with 15.0 mL of 1.0 M KOH aqueous solution. A constant current of 90 mA was applied between positive electrode and the negative electrode for 120 min.

2. Supplementary Results

2.1 Supplementary Figures

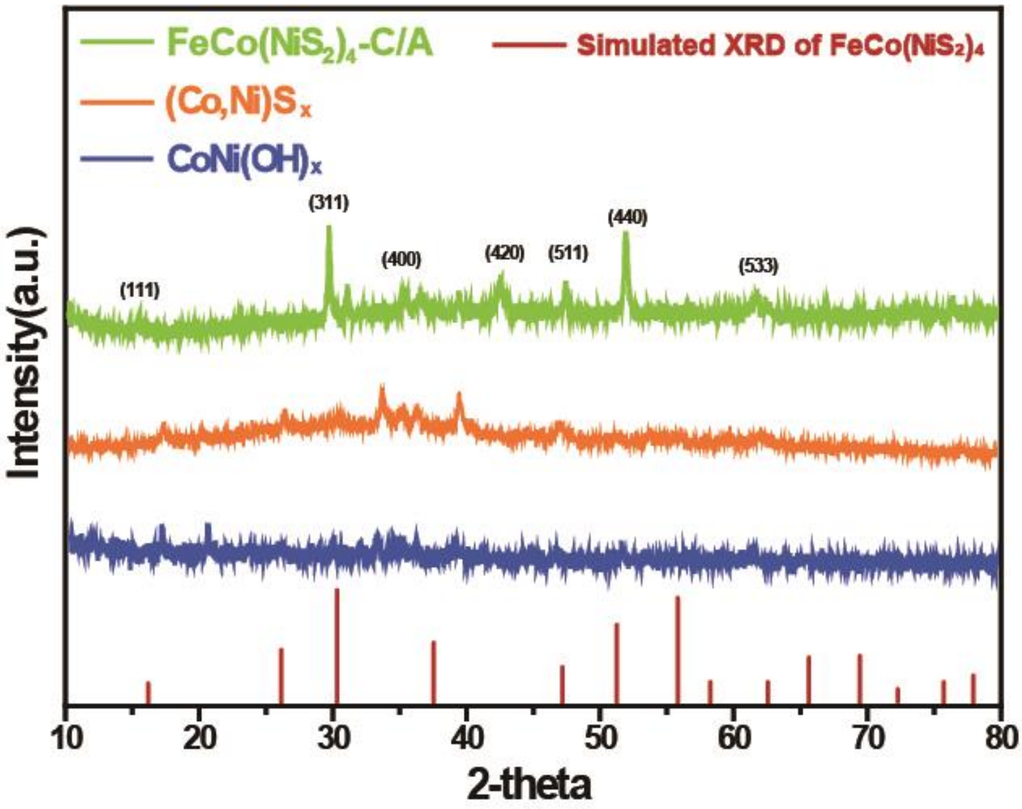

Figure S1. (a) XRD patterns of the $FeCo(NiS_2)_4$-C/A (cyan line), (Co, Ni)S_x (orange line) and CoNi$(OH)_x$ (blue line).

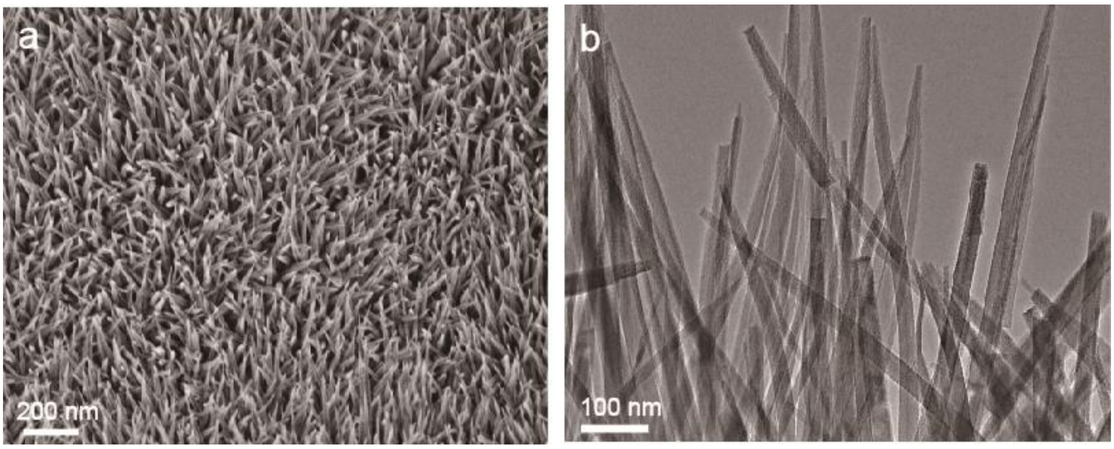

Figure S2. (a,b) SEM and (c ,d) TEM images of CoNi$(OH)_x$.

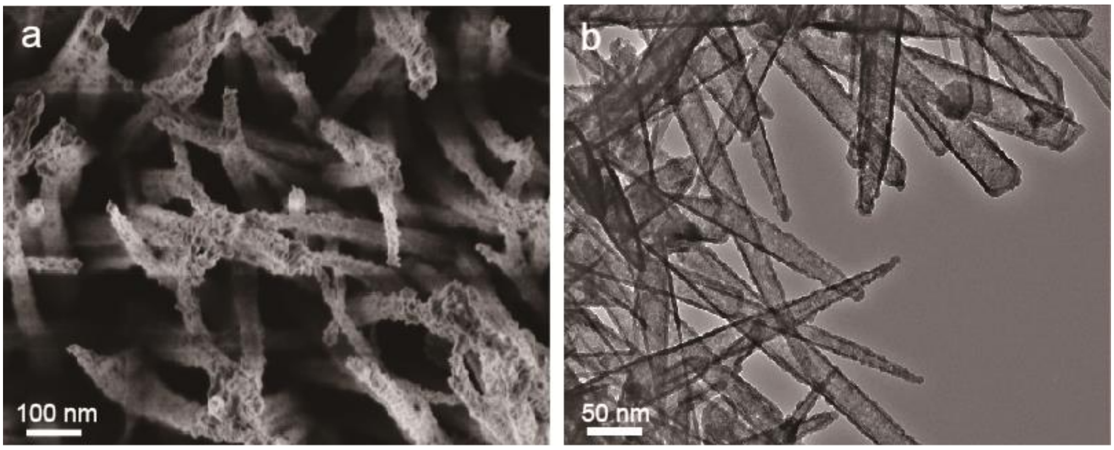

Figure S3. (a,b) SEM and (c ,d) TEM images of (Co,Ni)S_x.

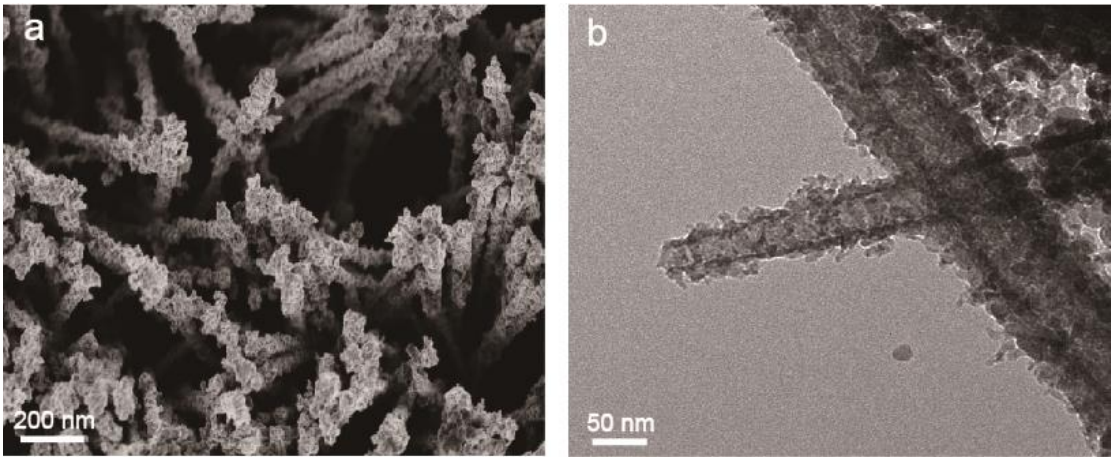

Figure S4. (a,b) SEM and TEM images of FeCo(NiS_2)$_4$-C/A.

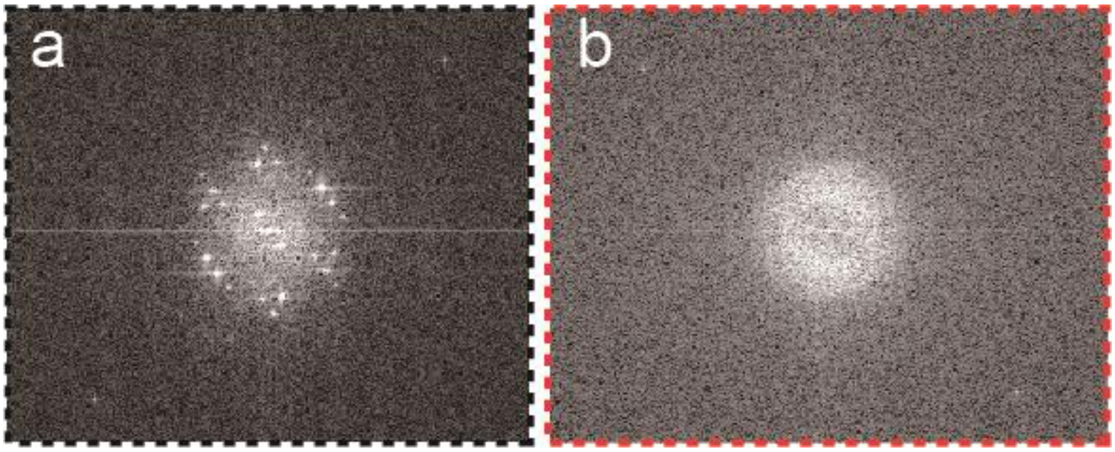

Figure S5. (a,b) The corresponding FFT patterns of the selected regions marked by the black and red squares from Figure 2b.

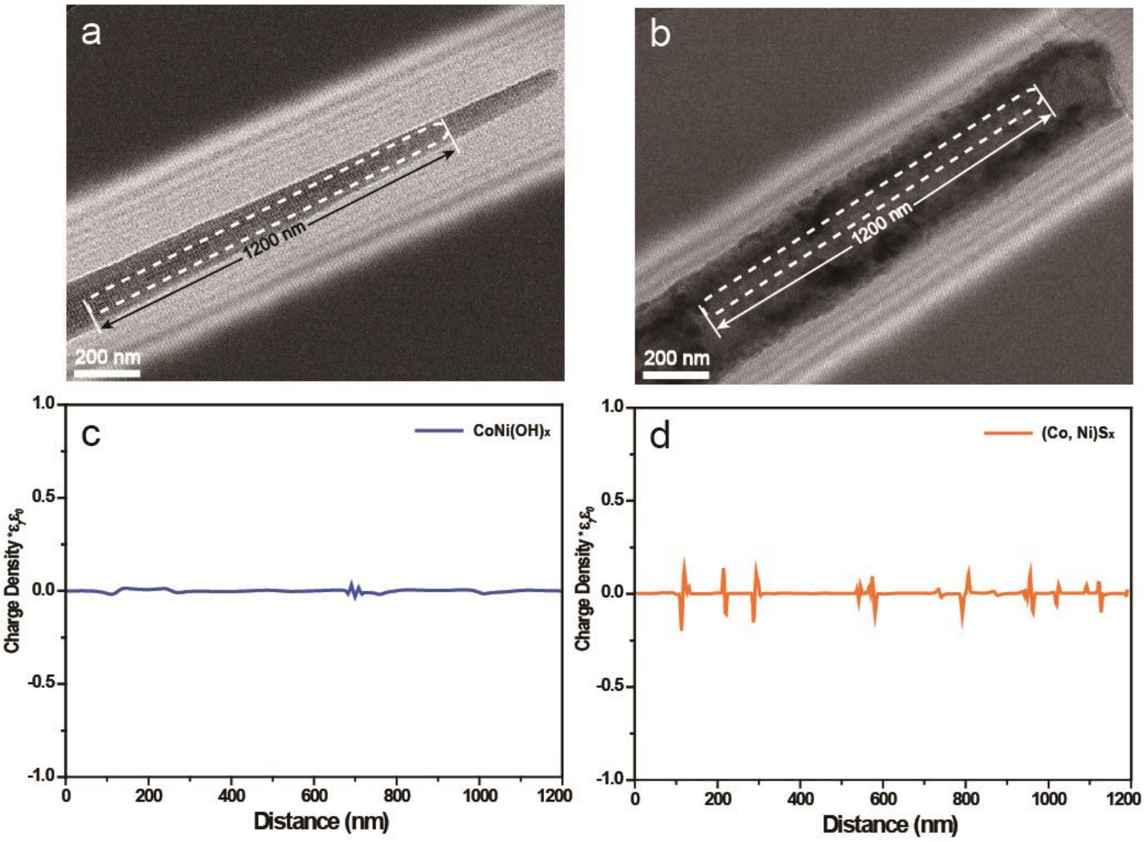

Figure S6. (a,b). Electron holography of (a) CoNi(OH)$_x$ and (b)(Co,Ni)S$_x$. (c,d) corresponding profiles for charge density distributions.

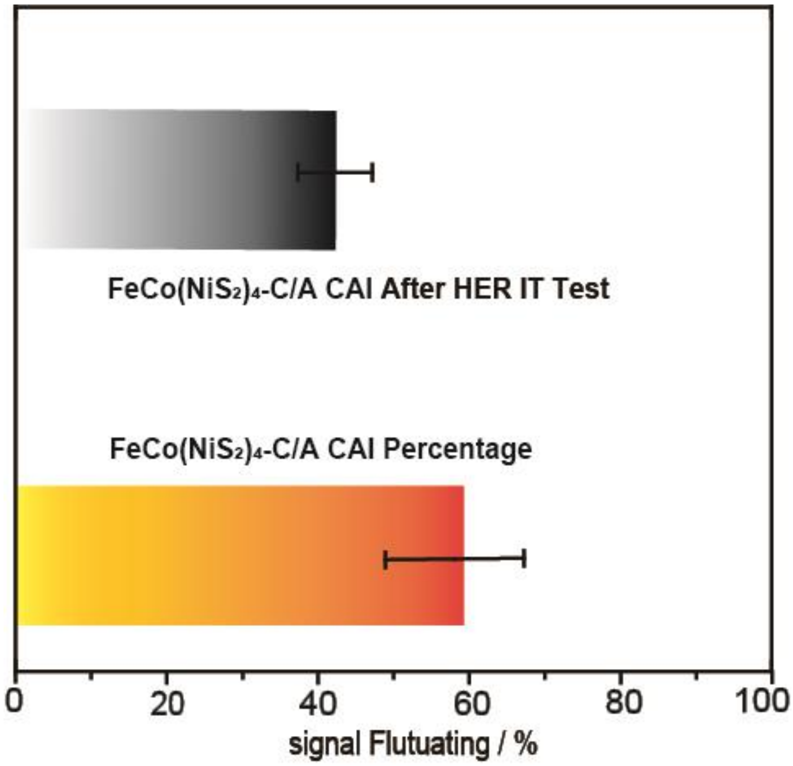

Figure S7. Statistics of signal fluctuations of charge densities for FeCo(NiS$_2$)$_4$-C/A and after IT test of FeCo(NiS$_2$)$_4$-C/A.

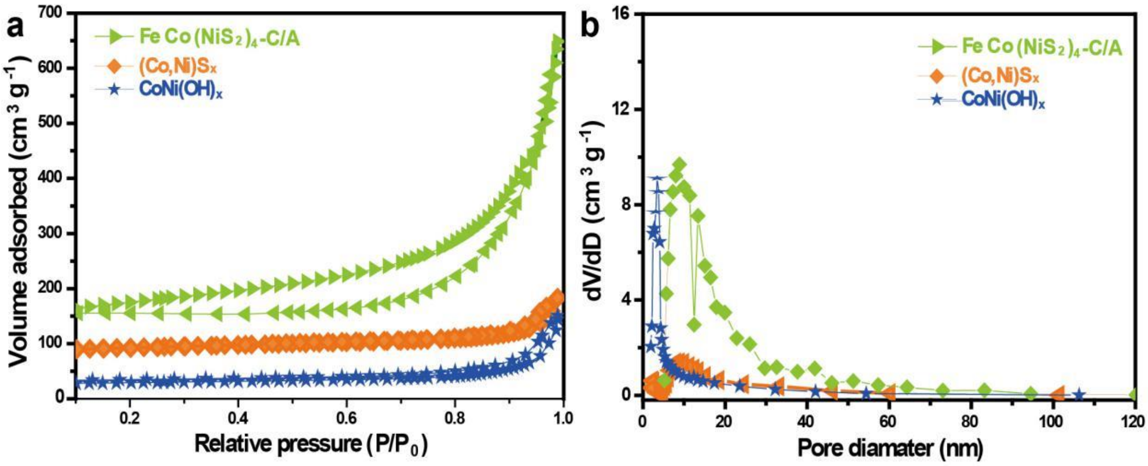

Figure S8. (a) N_2 adsorption-desorption isotherms, and (b) pore size distribution curves of the three samples.

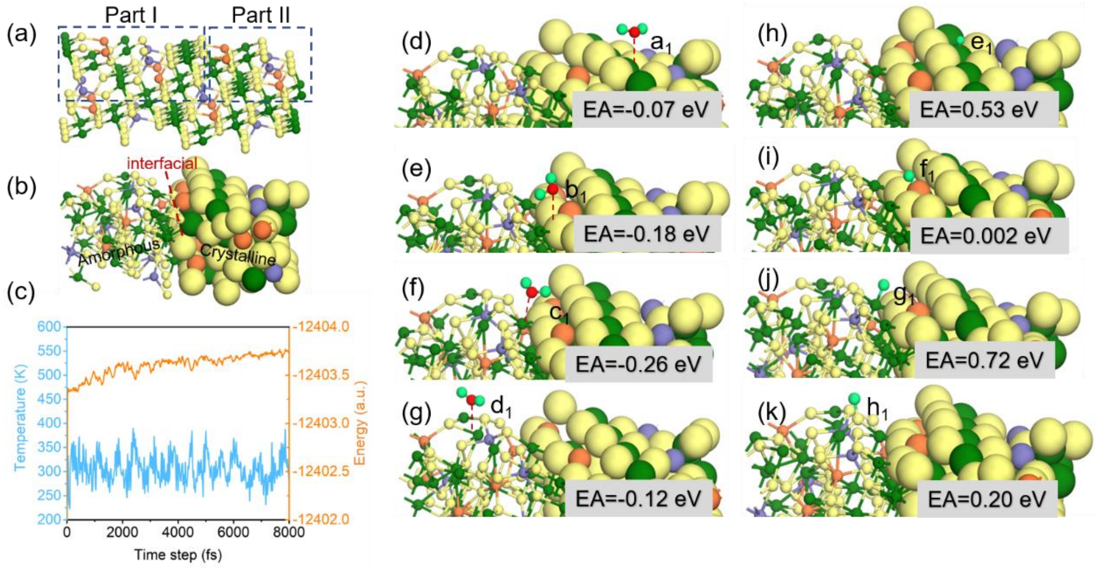

Figure S9. (a) The initial model of $FeCo(NiS_2)_4$-C/A heterostructure, where the sulfur vacancies were created in the part I. (b) The optimized $FeCo(NiS_2)_4$-C/A heterostructure after 10 ps AIMD simulations. (c) variations of temperatures and potential energies along with time steps for AIMD simulations of $FeCo(NiS_2)_4$-C/A heterostructure. (d-g) H_2O molecule adsorption on $FeCo(NiS_2)_4$-C/A heterostructure. (h-k) H atom adsorption configurations on $FeCo(NiS_2)_4$-C/A heterostructure. (l) The free energies diagrams of oxygen evolution reaction (OER) on $FeCo(NiS_2)_4$-C/A heterostructure.

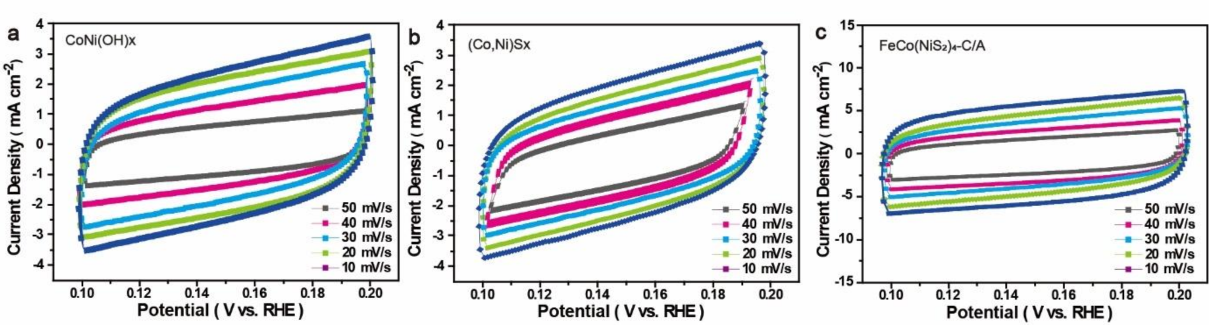

Figure S10. (a) CV curves of (a) $CoNi(OH)_x$, (b) $(Co,Ni)S_x$, (c) $FeCo(NiS_2)_4$-C/A electrocatalysts at scan rates from 10 to 50 mV s^{-1}.

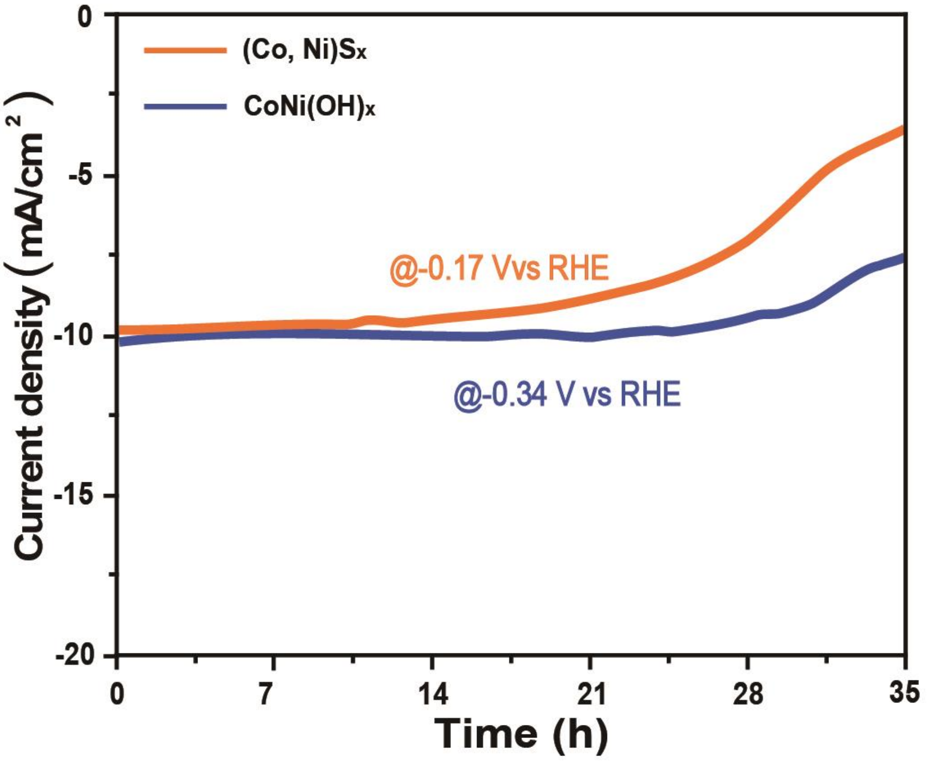

Figure S11. Long-term durability test of $CoNiS_x$ and $CoNi(OH)_x$ at different potentials for HER.

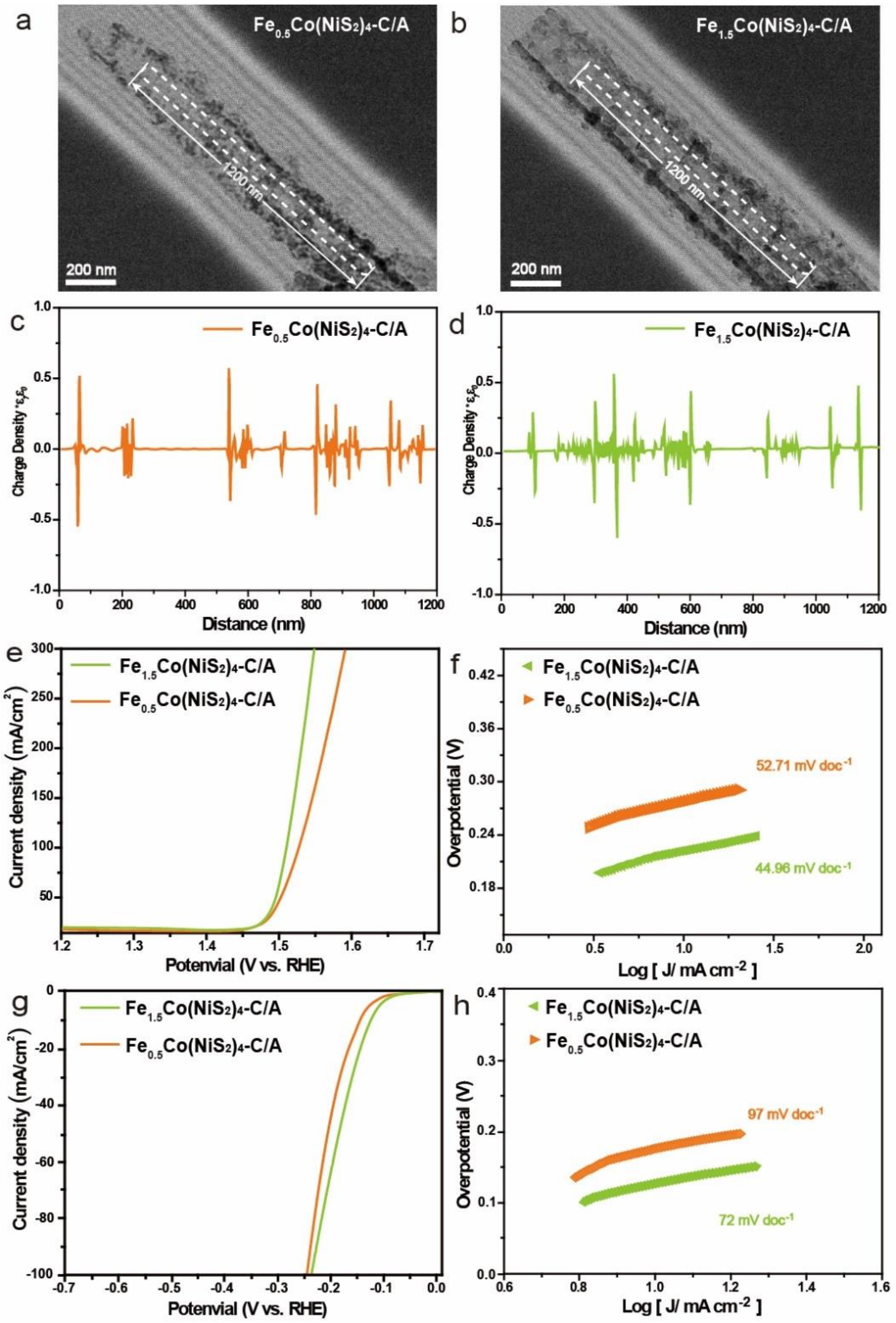

Figure S12. Electron holography of (a) $Fe_{0.5}Co(NiS_2)_4$-C/A and (b) $Fe_{1.5}Co(NiS_2)_4$-C/A, and corresponding charge density distribution of (c) $Fe_{0.5}Co(NiS_2)_4$-C/A and (d) $Fe_{1.5}Co(NiS_2)_4$-C/A. (e) OER performance: LSV curves of $Fe_{0.5}Co(NiS_2)_4$-C/A and $Fe_{1.5}Co(NiS_2)_4$-C/A in 1.0 m KOH solution. (f) Corresponding Tafel Slopes of $Fe_{0.5}Co(NiS_2)_4$-C/A and $Fe_{1.5}Co(NiS_2)_4$-C/A in terms of OER. (g) HER performance: LSV curves of $Fe_{0.5}Co(NiS_2)_4$-C/A and $Fe_{1.5}Co(NiS_2)_4$-C/A in 1.0 m KOH solution. (h) Corresponding Tafel Slopes of $Fe_{0.5}Co(NiS_2)_4$-C/A and $Fe_{1.5}Co(NiS_2)_4$-C/A in terms of HER.

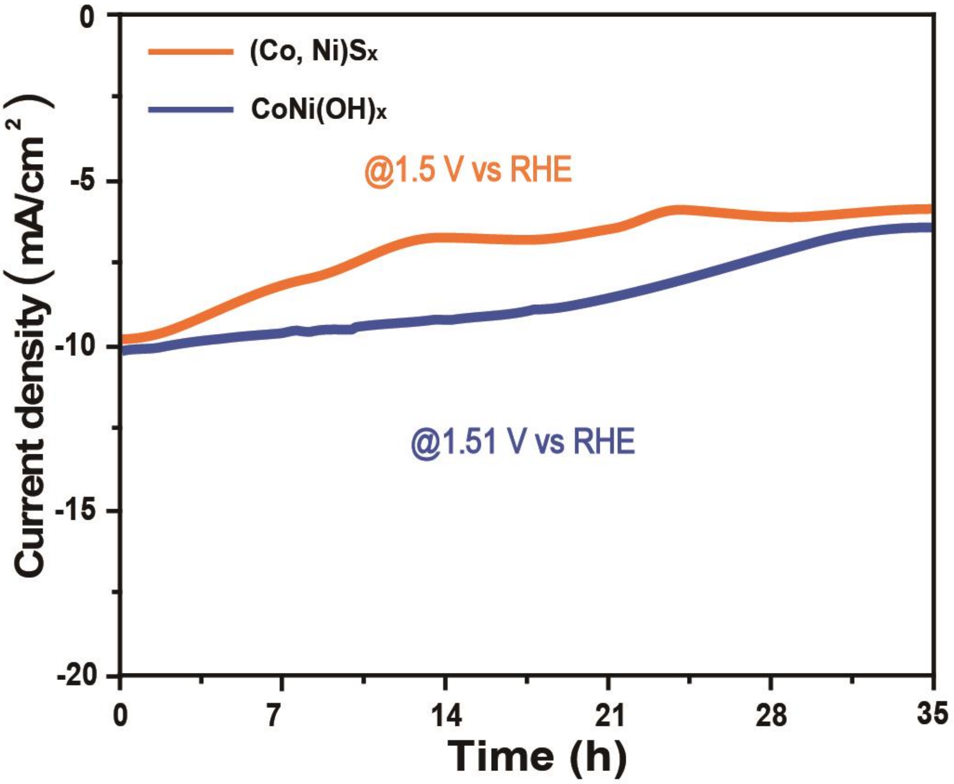

Figure S13. Long-term durability test of $CoNiS_x$ and $CoNi(OH)_x$ at different potentials for OER.

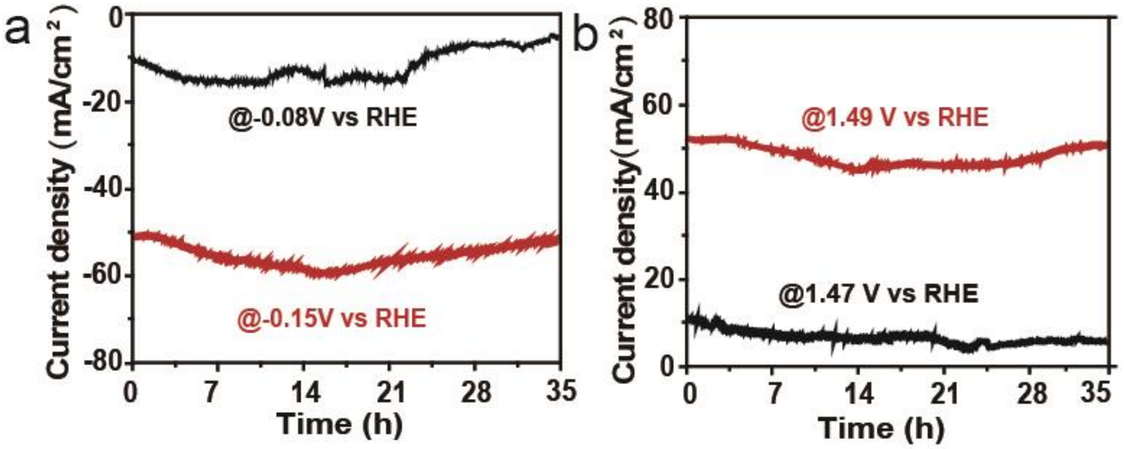

Figure S14. Long-term durability test of $FeCo(NiS_2)_4$-C/A at different potentials for HER and OER.

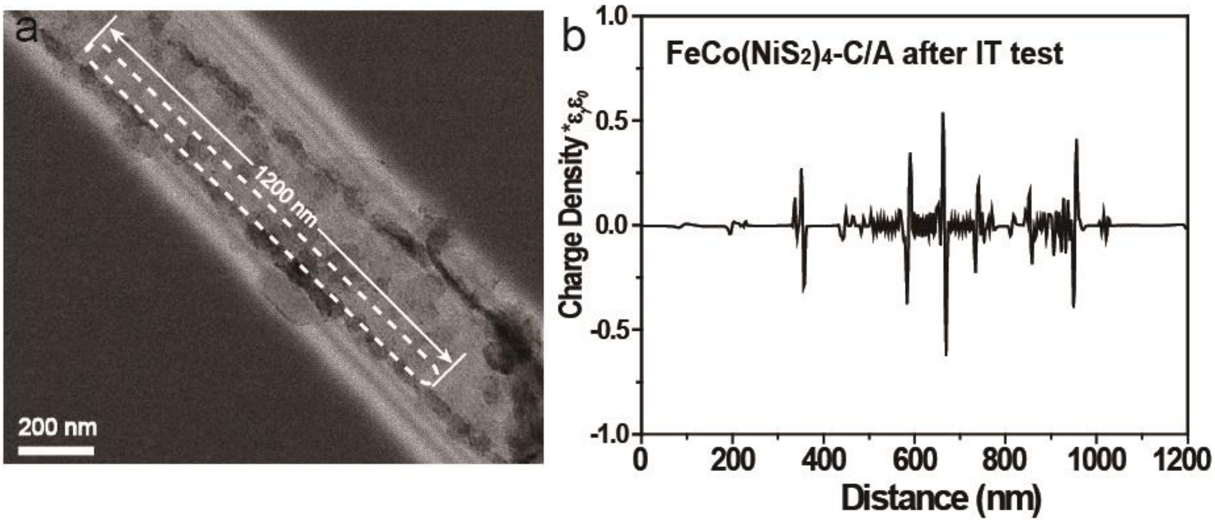

Figure S15. (a). Electron holography of FeCo$(NiS_2)_4$-C/A and (b)corresponding profiles for charge density distributions after IT test over 35 h.

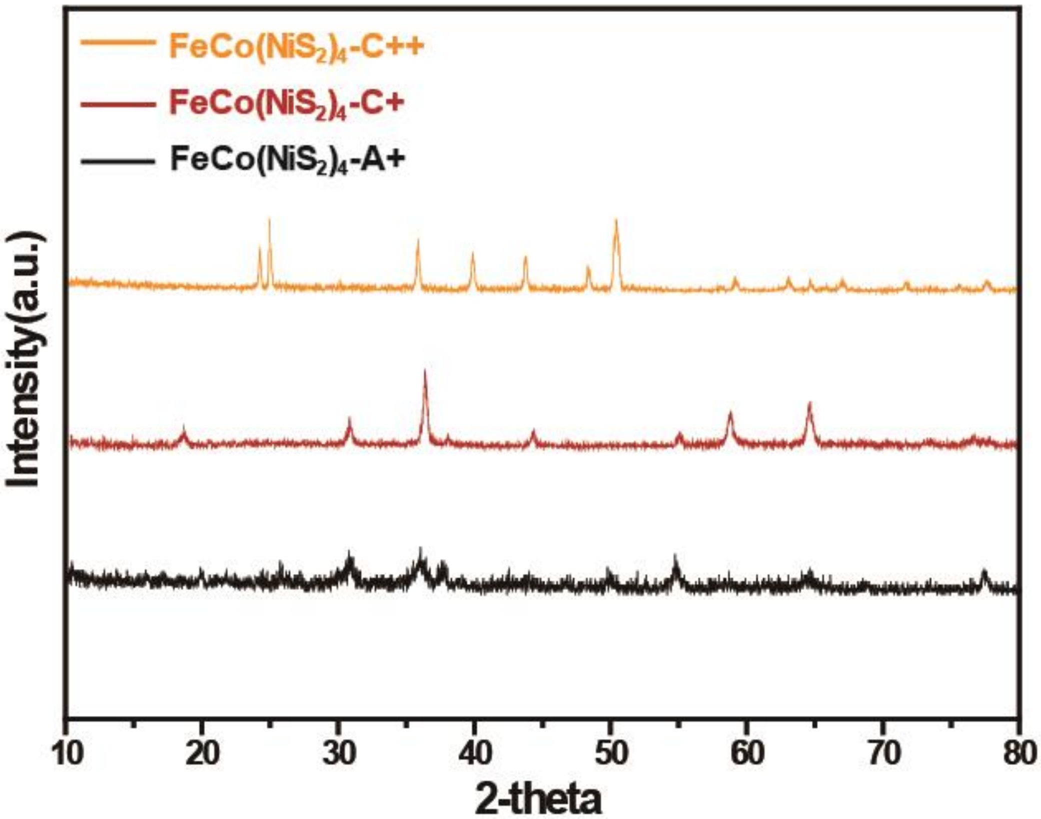

Figure S16. (a) XRD patterns of the FeCo$(NiS_2)_4$-C++ (yellow line), FeCo$(NiS_2)_4$-C+ (red line) and FeCo$(NiS_2)_4$-A+ (black line).

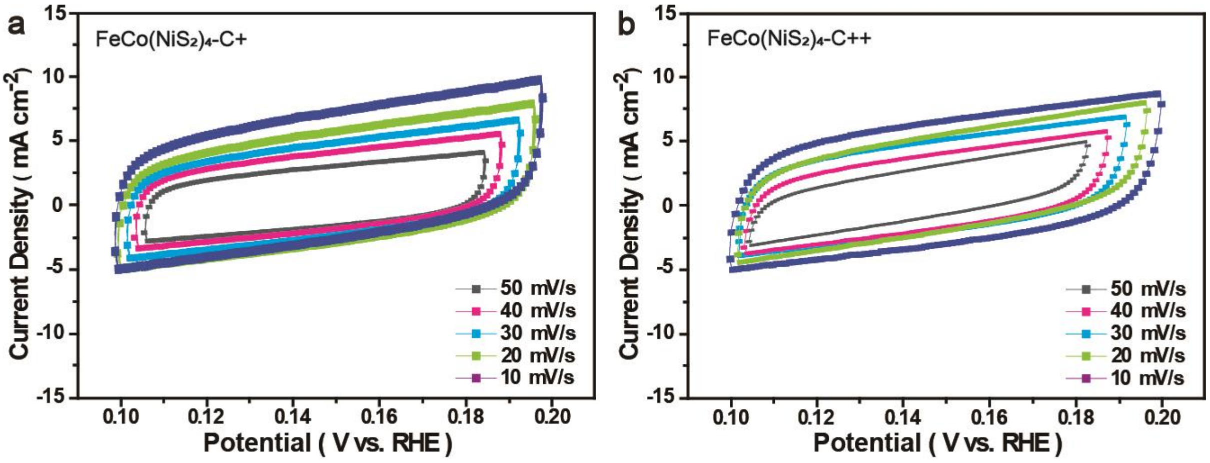

Figure S17. (a) CV curves of (a) FeCo(NiS_2)$_4$-C+, (b) FeCo(NiS_2)$_4$-C++ electrocatalysts at scan rates from 10 to 50 mV s^{-1}.

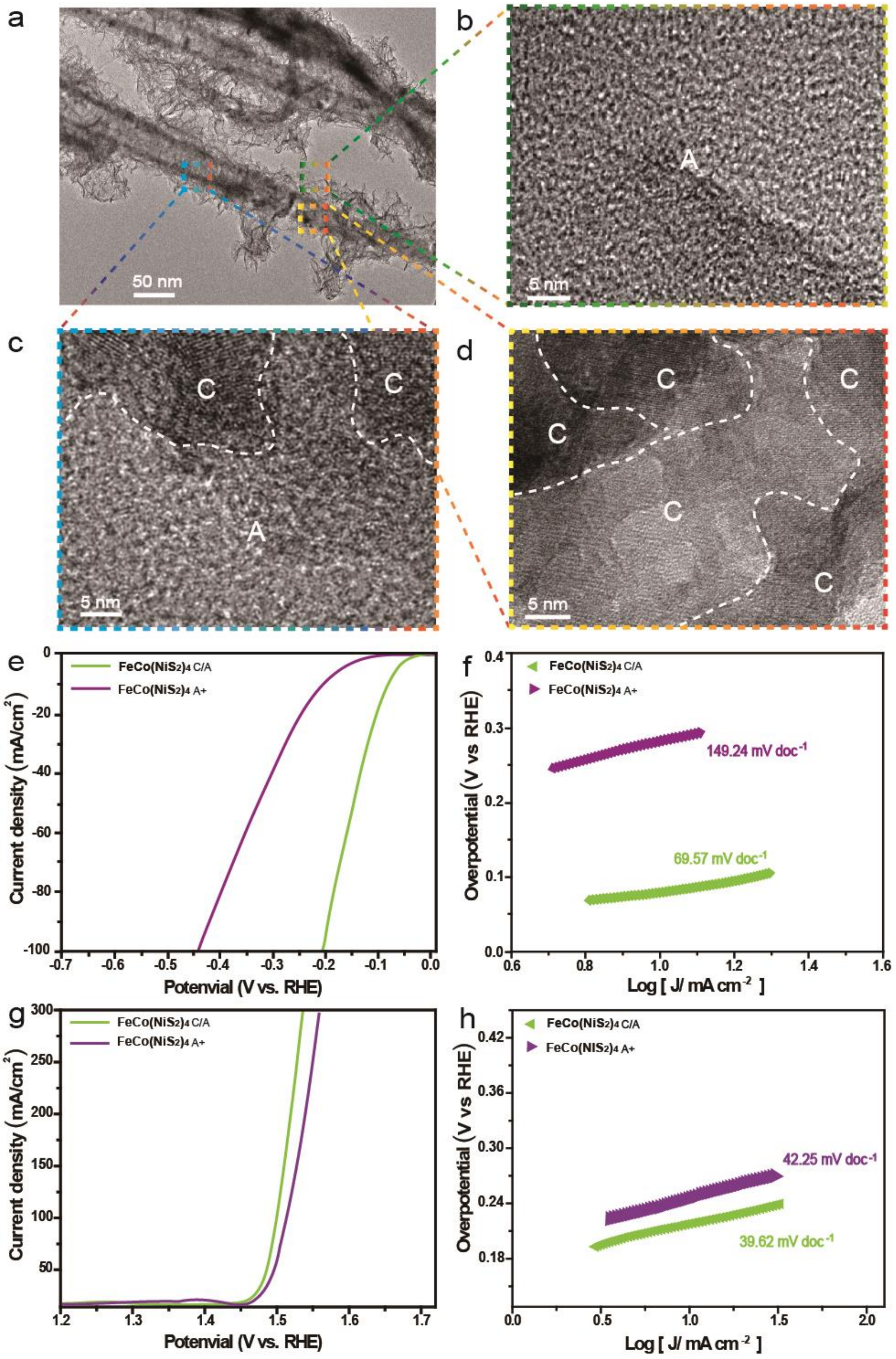

Figure S18. (a-d) TEM and HRTEM of as-prepared $FeCo(NiS_2)_4$-A^+. (e,f) LSV curves of different electrocatalysts in 1.0 m KOH solution and the corresponding HER Tafel plots. (g,h) LSV curves of different electrocatalysts in 1.0 m KOH solution and the corresponding OER Tafel plots.

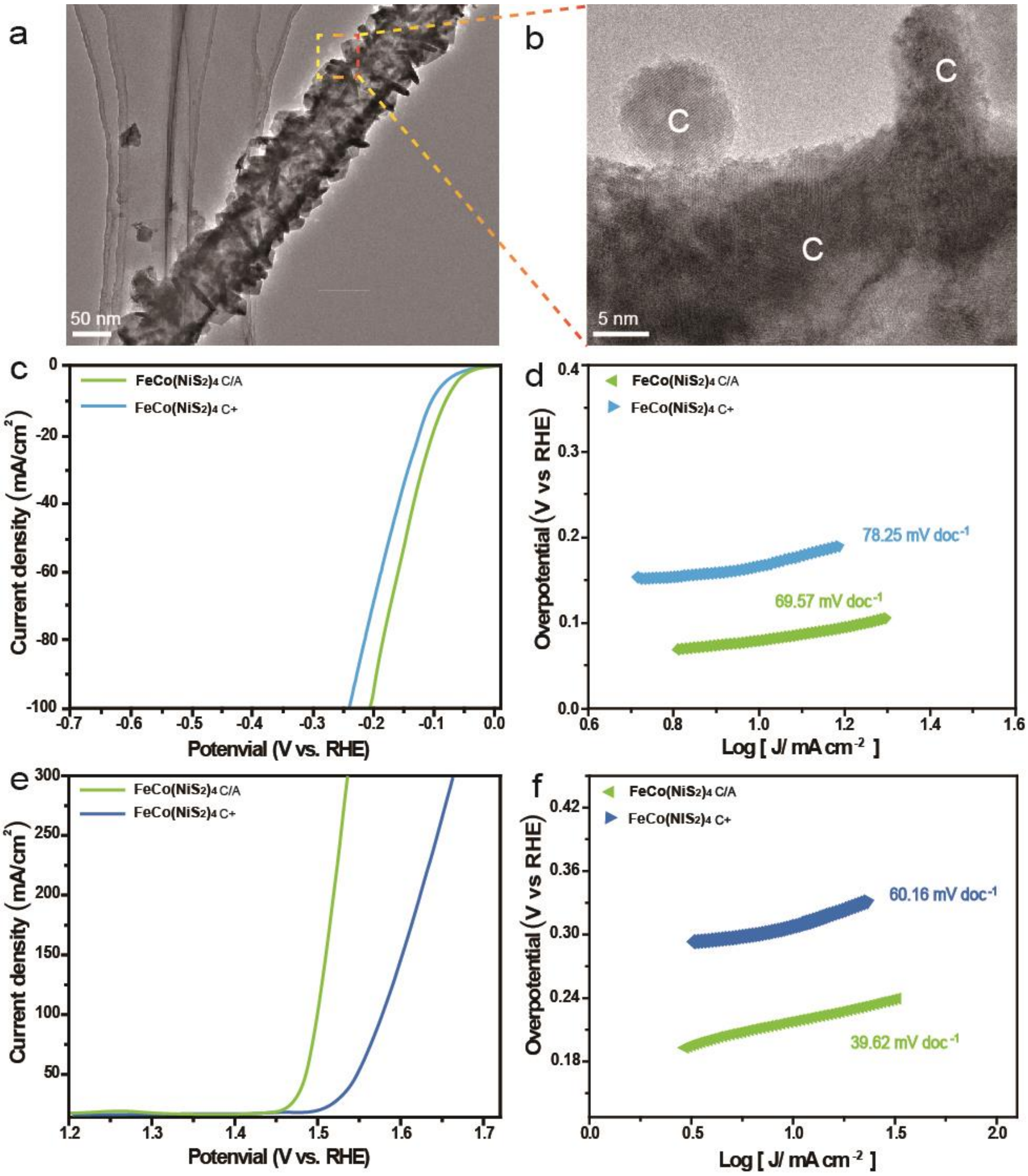

Figure S19. (a,b) TEM and HRTEM of as-prepared $FeCo(NiS_2)_4$-C^+. (c,d) LSV curves of different electrocatalysts in 1.0 m KOH solution and the corresponding HER Tafel plots. (e,f) LSV curves of different electrocatalysts in 1.0 m KOH solution and the corresponding OER Tafel plots.

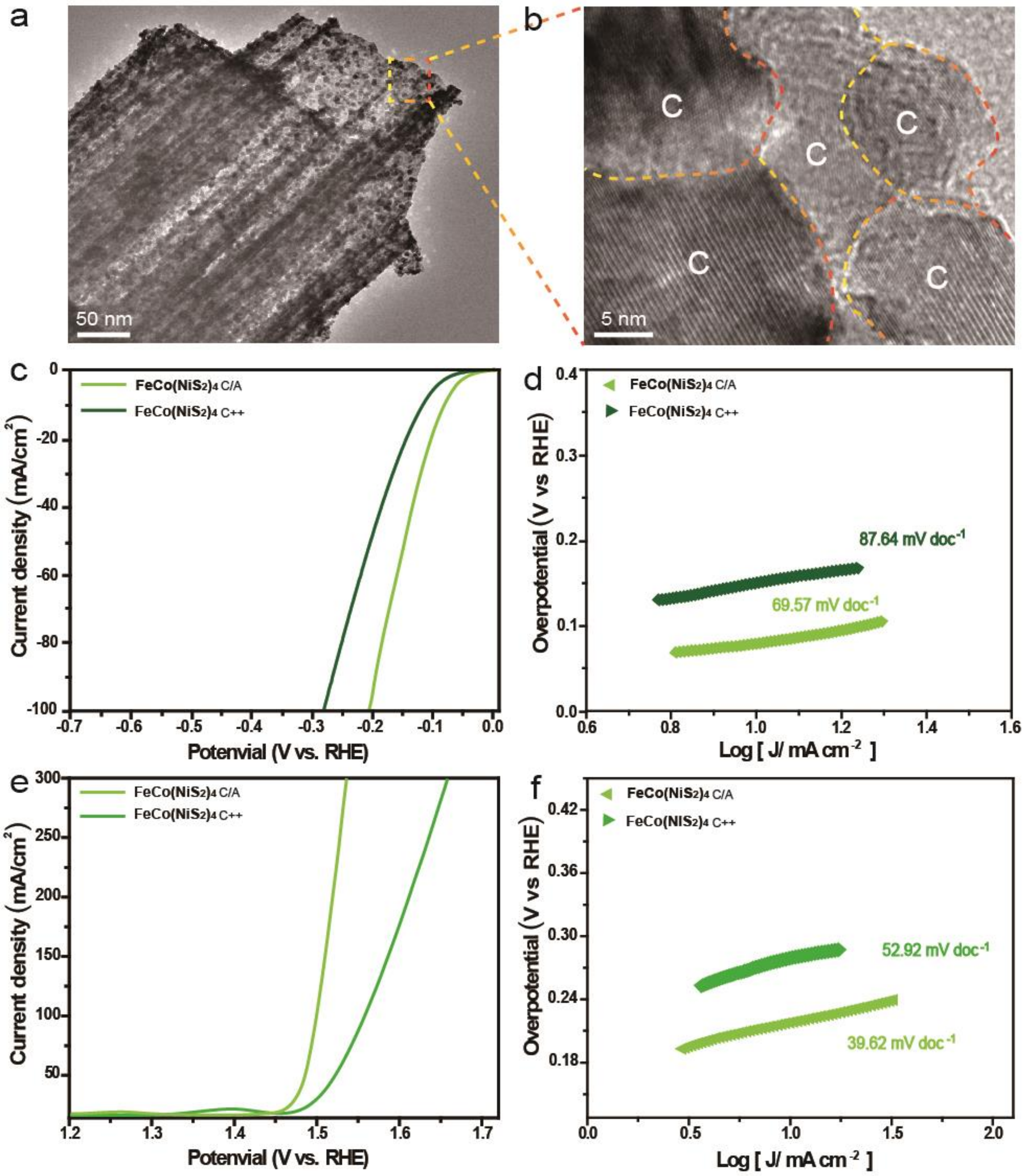

Figure S20. (a,b) TEM and HRTEM of as-prepared $FeCo(NiS_2)_4$-C^{++}. (c,d) LSV curves of different electrocatalysts in 1.0 m KOH solution and the corresponding HER Tafel plots. (e,f) LSV curves of different electrocatalysts in 1.0 m KOH solution and the corresponding OER Tafel plots.

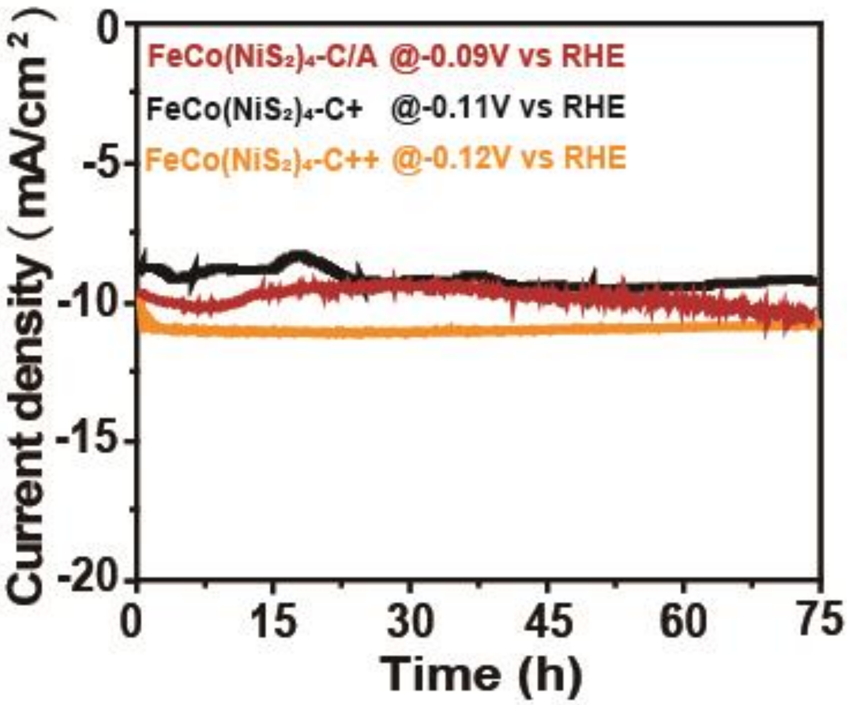

Figure S21. Long-term stability tests of $FeCo(NiS_2)_4$-C/A , $FeCo(NiS_2)_4$-C+ and $FeCo(NiS_2)_4$-C++ carried out at different potential using the same current density of 10 mA cm^{-2} for over 75 h.

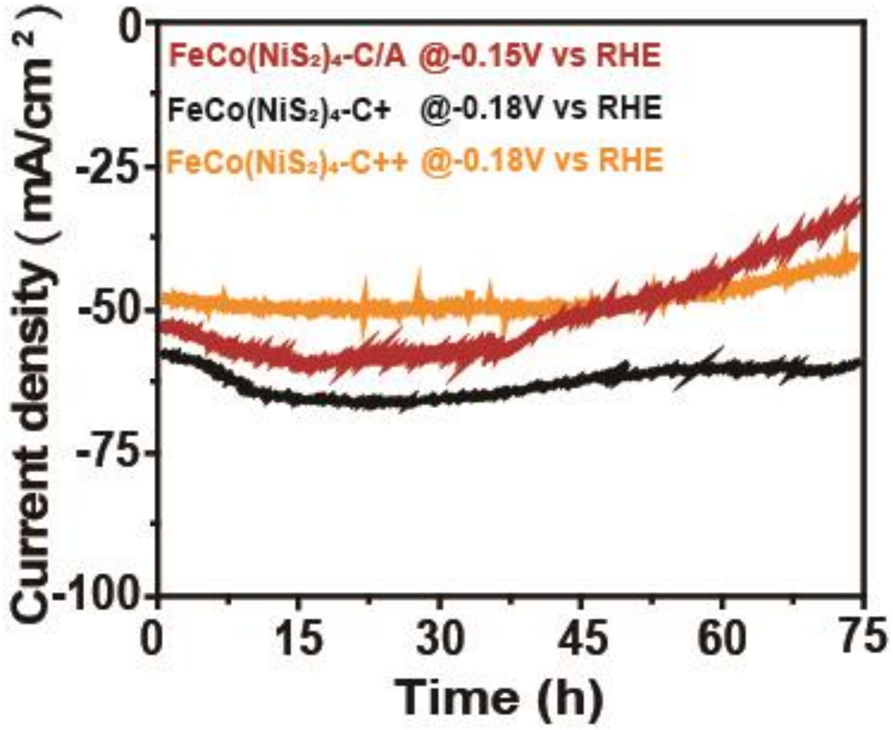

Figure S22. Long-term stability tests of $FeCo(NiS_2)_4$-C/A , $FeCo(NiS_2)_4$-C+ and $FeCo(NiS_2)_4$-C++ carried out at different potential using the same current density of 50 mA cm^{-2} for over 75 h.

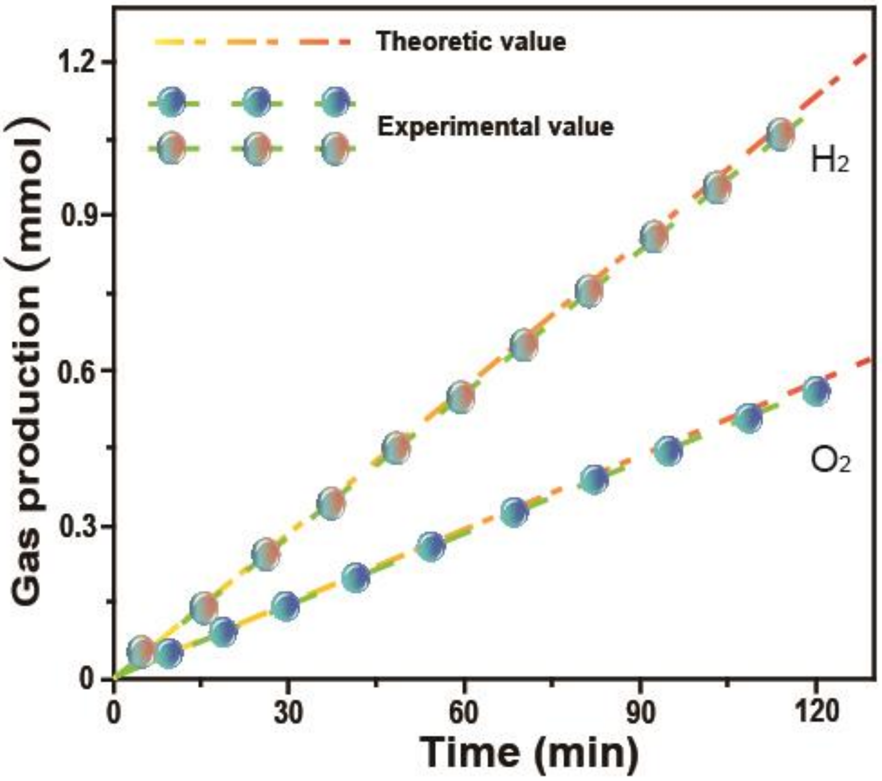

Figure S23. H_2 and O_2 production catalyzed by $CoFe(NiS_2)_4$-C/A in 1 M KOH at room temperature. The green dash lines present the theoretic value H_2 and O_2 amounts expected for a 100% Faradaic efficiency

2.2 Supplementary tables

Table S1. Comparison of the HER activities of the $FeCo(NiS_2)_4$-C/A sample in this work with recently-reported catalysts.

Catalysts	Overpotential (mV)	j /(mA cm^{-2})	Electrolyte	Reference
$FeCo(NiS_2)_4$-C/A	82	10	1.0 M KOH	**This work**
FeS_2/CoS_2	78.2	10	1.0 M KOH	S10
$MoS_2/Co_9S_8/$ Ni_3S_2/Ni	113	10	1.0 M KOH	S11
MoO_2-FeP	103	10	1.0 M KOH	S12
SAC-CuO NAs/CF	160	10	1.0 M KOH	S13
Cu_3P@NPPC	89	10	1.0 M H_2SO_4	S14
MoP@NiCo-LDH	454.3	10	1.0 M KOH	S15
Cu_3N-CuO	118	10	1.0 M KOH	S16
$MoSe_2$–Mo_2C	51	10	1.0 M KOH	S17
Pt-Cu@Cu_xO	72	10	1.0 M KOH	S18
NiCoMo	52	10	1.0 M KOH	S19

Table S2. Comparison of the OER activities of the $FeCo(NiS_2)_4$-C/A in this work with recently-reported catalysts.

**Catalysts**	**Overpotential (mV)**	**j /(mA cm^{-2})**	**Electrolyte**	**Reference**
$FeCo(NiS_2)_4$-C/A	230	10	1.0 M KOH	**This work**
FeS_2/CoS_2	302	10	1.0 M KOH	S10
$MoS_2/Co_9S_8/Ni_3S_2/Ni$	166	10	1.0 M KOH	S11
Fe_3O_4/FeS_2	253	10	1.0 M KOH	S20
$Cu_2SCu(OH)_2$/CF	268	10	1.0 M KOH	S21
FeNi-LDH/CoP	231	10	1.0 M KOH	S22
$NiSe_2/CoSe_2$	286	10	1.0 M KOH	S23
$NiCo_{16-x}P_6$	290	10	1.0 M KOH	S24
CoP-CeO_2	224	10	1.0 M KOH	S25
Pt-Cu@Cu_xO	250	10	1.0 M KOH	S18
NiCoMo	304	10	1.0 M KOH	S19

Table S3. Comparison of over water splitting performance of $FeCo(NiS_2)_4$-C/A in this work with recently-reported catalysts.

Catalysts	Overpotential (mV)	j /(mA cm^{-2})	Electrolyte	Reference
$FeCo(NiS_2)_4$-C/A	1.51	10	1.0 M KOH	**This work**
FeS_2/CoS_2	1.47	10	1.0 M KOH	S10
$MoS_2/Co_9S_8/$ Ni_3S_2/Ni	1.54	10	1.0 M KOH	S11
Pt-CoS_2/CC	1.55	10	1.0 M KOH	S26
CoP–N/Co foam	1.61	10	1.0 M KOH	S27
$CoNiMoO_4$-21/CuO_x/CF	1.532	10	1.0 M KOH	S28
Cu_3N-CuO	1.62	10	1.0 M KOH	S16
SAC-CuO NAs/CF	1.51	10	1.0 M KOH	S13
MoP@NiCo LDH	1.697	20	1.0 M KOH	S15
RuO_2/C ‖ Pt/C	1.70	10	1.0 M KOH	S29
NiCoMo	304	10	1.0 M KOH	S19

References

S1. Y. Yang, L. Dang, M. J. Shearer, H. Sheng, W. Li, J. Chen, P. Xiao, Y. Zhang, R. J. Hamers, S. Jin, *Adv. Energy Mater.* **2018**, *8*, 1703189.

S2. S. A. Mohammad, K. Haekyoung, *Fuel* **2021**, *304*, 121309

S3. J. VandeVondele, M. Krack, F. Mohamed, M. Parrinello, T. Chassaing, J. Hutter, *Comput. Phys. Commun.* **2005**, *167*, 103–128.

S4. S. Goedecker, M. Teter, J. Hutter, *Phys. Rev. B* **1996**, *54*, 1703.

S5. J. VandeVondele, J. Hutter, *J. Chem. Phys.* **2007**, *127*, 114105.

S6. G. Kresse, J. Furthmüller, *Phys. Rev. B* **1996**, *54*, 11169.

S7. J. P. Perdew, K. Burke, M. Ernzerhof, *Phys. Rev. Lett.* **1996**, *77*, 3865.

S8. S. Ehrlich, J. Moellmann, W. Reckien, T. Bredow, S. Grimme, *ChemPhysChem* **2011**, *12*, 3414-3420.

S9. J. K. Norskov, J. Rossmeisl, A. Logadottir, L. Lindqvist, J. R. Kitchin, T. Bligaard, H. Jonsson, *J. Phys. Chem. B* **2004**, *108*, 17886–17892.

S10. Y. Li, J. Yin, L. An, M. Lu, K. Sun, Y-Q. Zhao, D. Gao, F. Cheng, P. Xi, *Small*. **2018**, *14*, 1801070.

S11. Y. Yang, H. Yao, Z. Yu, S. M. Islam, H. He, M. Yuan, Y. Yue, K. Xu, W. Hao, G. Sun, H. Li, S. Ma, P. Zapol, M. G. Kanatzidis, *J. Am. Chem. Soc.* **2019**, *141*, 10417–10430.

S12. G. Yang, Y. Jiao, H. Yan, Y. Xie, A. Wu, X. Dong, D. Guo, C.Tian, H. Fu, *Adv. Mater.* **2020**, *32*,2000455.

S13. H. Xu, T. Liu, S. Bai, L. Li, Y. Zhu, J. Wang, S. Yang, Y. Li, Q. Shao, X. Huang, *Nano Lett.* **2020**, *20*, 5482–5489.

S14. R. Wang, X-Y. Dong, J. Du, J-Y, Zhao, S-Q. Zang, *Adv. Mater.* **2018**, *30*,1703711.

S15. T. Wang, H. Wu, C. Feng, L. Zhang, J. Zhang, *J. Mater. Chem. A* **2020**, *8*, 18106–18116.

S16. C. Panda, P. W. Menezes, M. Zheng, S. Orthmann, M. Driess, *ACS Energy Lett*. **2019**, *4*,747–754.

S17. J. Li, W. Hong, C. Jian, Q. Cai, X. He, W. Liu, *J. Mater. Chem. A* **2020**, *8*, 6692–6698.

S18. D. T. Tran, H. T. Le, T. L. L. Doan, N. H. Kim, J. H. Lee, *Nano Energy*. **2019**, *59*, 216–228.

S19. S. Hao, L. Chen, C. Yu, B. Yang, Z. Li, Y. Hou, L. Lei, X. Zhang, *ACS Energy Lett.* **2019**, *4*,952–959.

S20. M. J. Wang, X. Zheng, L. Song, X. Feng, Q. Liao, J. Li, L. Li, Z. Wei, *J. Mater. Chem. A* **2020**,*8*, 14145–14151.

S21. D. Wang, J. Li, Y. Zhao, H.Xu, J. Zhao, *Electrochim. Acta.* **2019**, *316*, 8–18.

S22. K. He,T. T. Tsega, X. Liu, J. Zai, X.-H. Li, X. Liu,W. Li, N. Ali, X. Qian, *Angew. Chem. Int. Ed.* **2019**, *58*, 11903–11909.

S23. X. Zheng, X. Han,Y. Cao,Y. Zhang, D. Nordlund, J. Wang, S. Chou, H.Liu, L. Li, C. Zhong,Y. Deng, W. Hu, *Adv. Mater.* **2020**, *32*, 2000607.

S24. Y. Zhao, J. Zhang, Y. Xie, B. Sun, J. Jiang, W.-J. Jiang, S. Xi, H.Y. Yang, K. Yan, S. Wang, X. Guo, P. Li, Z. Han, X. Lu, H. Liu and G. Wang, *Nano Lett.* **2021**, *21*, 823.

S25. M. Li, X. Pan, M. Jiang, Y. Zhang, Y. Tang and G. Fu, *Chem. Eng. J.* **2020**, *395*, 125160.

S26. X. Han, X. Wu, Y. Deng, J. Liu, J. Lu, C. Zhong, W. Hu, *Adv. Energy Mater.* **2018**,1800935.

S27. Z. Liu, X. Yu, H. Xue, L. Feng, *J. Mater. Chem. A* **2019**, *7*, 13242–13248.

S28. M. Gu, X. Deng, M. Lin, H. Wang, A. Gao, X. Huang, Xiaojun Zhang, *Adv. Energy Mater.* **2021**, *11*, 2102361

S29. B. Zhang, C. Zhu, Z. Wu, E. Stavitski, Y. H. Lui, T. Kim, H. Liu, L.Huang, X. Luan, L. Zhou, K. Jiang, W. Huang, S. Hu, H. Wang, J. S. Francisco, *Nano Lett.* **2020**, *20*, 136–144.
